# Scaling up overdose education and naloxone distribution in Kentucky: adoption and reach achieved through a “hub with many spokes” model

**DOI:** 10.1186/s13722-023-00426-6

**Published:** 2023-11-30

**Authors:** Hannah K. Knudsen, Patricia R. Freeman, Douglas R. Oyler, Carrie B. Oser, Sharon L. Walsh

**Affiliations:** 1 Department of Behavioral Science and Center on Drug & Alcohol Research, University of Kentucky, 845 Angliana Avenue, Room 204, Lexington, KY 40508 USA; 2 Department of Pharmacy Practice and Science and Center for the Advancement of Pharmacy Practice, College of Pharmacy, University of Kentucky, Lexington, KY 40536 USA; 3 Department of Pharmacy Practice and Science, College of Pharmacy, University of Kentucky, Lexington, KY 40536 USA; 4 Department of Sociology, Center on Drug & Alcohol Research, and Center for Health Equity Transformation, University of Kentucky, Lexington, KY 40536 USA

**Keywords:** Naloxone, Overdose education and naloxone distribution, Implementation strategies, Opioid overdose, Opioid epidemic

## Abstract

**Background:**

Scaling up overdose education and naloxone distribution (OEND), an evidence-based practice for reducing opioid overdose mortality, in communities remains a challenge. Novel models and intentional implementation strategies are needed. Drawing upon the EPIS model’s phases of Exploration, Preparation, Implementation, and Sustainment (Aarons et al. in Adm Policy Ment Health 38:4–23, 2011), this paper describes the development of the University of Kentucky’s unique centralized “Naloxone Hub with Many Spokes” approach to implementing OEND as part of the HEALing Communities Study (HCS-KY).

**Methods:**

To scale up OEND in eight Kentucky counties, implementation strategies were utilized at two levels: a centralized university-based naloxone dispensing unit (“Naloxone Hub”) and adopting organizations (“Many Spokes”). Implementation strategies varied across the EPIS phases, but heavily emphasized implementation facilitation. The Naloxone Hub provided technical assistance, overdose education resources, and no-cost naloxone to partner organizations. Implementation outcomes across the EPIS phases were measured using data from internal study management trackers and naloxone distribution data submitted by partner organizations.

**Results:**

Of 209 organizations identified as potential partners, 84.7% (n = 177) engaged in the Exploration/Preparation phase by participating in an initial meeting with an Implementation Facilitator about the HCS-KY OEND program. Adoption of the HCS-KY OEND program, defined as receipt of at least one shipment of naloxone, was achieved with 69.4% (n = 145) of all organizations contacted. During the Implementation phase, partner organizations distributed 40,822 units of naloxone, with partner organizations distributing a mean of 281.5 units of naloxone (SD = 806.2). The mean number of units distributed per county was 5102.8 (SD = 3653.3; range = 1057 − 11,053) and the mean county level distribution rate was 8396.5 units per 100,000 residents (SD = 8103.1; range = 1709.5–25,296.3). Of the partner organizations that adopted the HCS-KY OEND program, 87.6% (n = 127) attended a sustainability meeting with an Implementation Facilitator and agreed to transition to the state-funded naloxone program.

**Conclusions:**

These data demonstrate the feasibility of this “Hub with Many Spokes” model for scaling up OEND in communities highly affected by the opioid epidemic.

*Trial registration* ClinicalTrials.gov, NCT04111939. Registered 30 September 2019, https://clinicaltrials.gov/ct2/show/NCT04111939.

**Supplementary Information:**

The online version contains supplementary material available at 10.1186/s13722-023-00426-6.

## Background

The United States (US) opioid epidemic has exacted a massive toll on individuals, families, and communities, and, although the number of overdose deaths may be leveling off, data from 2021 indicates 109,000 Americans died from drug overdose, with the majority of deaths from synthetic opioids [1]. Costs of the opioid epidemic likely exceed $1 trillion due to a combination of premature mortality, lost productivity, healthcare to treat opioid use disorder (OUD) and its medical consequences, and criminal justice expenditures [2]. Kentucky has been particularly hard hit, with a 45% increase in opioid overdose deaths from 2019 to 2020 [3], a rate of 55.6 deaths per 100,000 residents in 2021 [4], and a current ranking as the state with the fourth highest fatal drug overdoses in the US. [5]. During the early phase of the COVID-19 pandemic, Kentucky was noted as one of the states with the greatest magnitude increase in opioid overdose deaths, with 56 monthly deaths per million residents in May 2020 [6]. Given the ongoing number of lives lost, there continues to be an urgent need to scale up evidence-based practices (EBP) that can mitigate and alleviate the burden of the opioid crisis.

Overdose education and naloxone distribution (OEND) is a highly effective EBP to reduce opioid-related mortality. Self-report data from OEND recipients consistently demonstrates that naloxone administration results in survival in > 90% of cases [7–10]. One recent study indicated that bystander naloxone administration during an overdose produces an eight-fold increase in the odds of survival [11]. Overdose education, which can be relatively brief in duration, increases bystander knowledge about recognizing and appropriately responding to an overdose [7, 10, 12]. Although some community members may be concerned that OEND may represent a “moral hazard,” a recent systematic review found that OEND does not increase substance use [13].

At the community level, scaling up OEND by increasing naloxone distribution within communities is a powerful strategy for reducing opioid overdose deaths. Observational studies have shown that communities with greater naloxone saturation (i.e., more population-adjusted units distributed) have lower opioid overdose mortality than communities with lower saturation [14, 15]. Modeling studies indicate that OEND is the most impactful EBP for scaling up to reduce overdose deaths [16–18], particularly when fentanyl is the dominant opioid in the community [19]. Economic models estimate that each $1 investment in scaling up OEND leads to savings of more than $2700 due to decreasing premature mortality [14].

Despite evidence that scaling up OEND would be highly impactful, access to OEND remains challenging in many communities. There are several models of OEND that communities could scale up. These include encouraging prescriptions directly from medical providers, implementing standing orders at pharmacies, or increasing community distribution programs (i.e., “take-home naloxone programs”) [19]. Depending on state regulations, community distribution may rely on a statewide standing order, or programs may partner with a medical provider who signs a standing order authorizing the program to distribute naloxone on their behalf. However, access to OEND remains sub-optimal. For example, harm reduction programs, such as syringe service programs (SSPs) are an important site for community distribution [14], and nearly all SSPs report that they distribute naloxone [20]. However, just 26% of U.S. SSPs account for 81% of the units of naloxone distributed through this venue [20], suggesting concentration effects such that many SSPs may not be fully meeting their community’s needs. Pharmacies can play an important role in OEND, but a recent audit study reported that only about 70% of pharmacies in 11 states had naloxone available [21]. A study of opioid-related emergency department visits demonstrated that only 1.1.% of individuals filled a naloxone prescription within 30 days of the visit [22]. Baseline data from an observational study of implementing OEND in an opioid treatment program indicated that less than 5% of patients had received a prescription for naloxone in the prior year [23]. Furthermore, survey data from US laypersons indicates that < 1% had received naloxone [24].

Implementation science may represent a useful lens for considering how to scale up OEND in communities [25], as it offers frameworks for (1) examining how organizational and environmental contexts may represent facilitators or barriers to the phases of the implementation process [26–29], (2) measuring outcomes [30, 31], and (3) intentionally deploying implementation strategies to increase the likelihood of implementation success [32, 33]. Some research has drawn on concepts from implementation science to identify barriers to OEND implementation, such as cost and billing challenges, stigma among leadership and staff, workflow and logistical challenges, lack of training resources, limited access to medical providers to sign standing order agreements, transportation barriers experienced by patients to travel to pharmacies, and confusion over state regulations regarding OEND [34–39].

Other U.S. studies have used implementation outcomes (e.g., reach, adoption) to describe OEND efforts, but typically only within a single setting or system. For example, Devries et al. [40] described OEND implementation in a single university health system, where implementation strategies included dissemination of clinical guidelines and patient educational materials, pharmacy stocking of naloxone, and provider training, which resulted in 245 naloxone prescriptions over a 10-month period (i.e., the implementation outcome of reach). National scale-up of OEND within the Veterans Administration health system relied on implementation strategies of a website with implementation planning tools, clinical guidelines and provider training, standardized “kits” with naloxone and patient education materials, addition of naloxone to the national formulary, stocking of naloxone in the mail order pharmacy and on-site outpatient pharmacies, and academic detailing (i.e., short in-person educational visits made by pharmacists) to providers to encourage naloxone prescribing [41]. Over a 3-year period, nearly 5700 providers wrote at least one prescription (representing the outcome of provider adoption) with reach of more than 45,000 naloxone prescriptions [41]. Notably, the implementation strategy of academic detailing enhanced the reach of OEND, such that providers receiving academic detailing achieved greater naloxone prescription rates than those who did not receive academic detailing [42].

Although studies of health systems offer insight into scaling up OEND, it is less clear about which implementation strategies are needed to scale up OEND in diverse organizational settings and sectors. Two international studies offer notable examples of multi-sectoral OEND scale-up in communities. In Alberta Canada, a single province-wide health authority partnered with organizations representing harm reduction, emergency departments, walk-in clinics, corrections, pharmacies, treatment, and colleges to scale up OEND [43]. Key implementation strategies included identifying funding; changing policy over time to allow additional medical professionals to prescribe naloxone, ultimately rescheduling naloxone so that a prescription was not required; and developing online provider education resources. Over a 1-year period, 759 pharmacies agreed to dispense naloxone and 194 other locations adopted OEND, resulting in a reach of 9572 units of naloxone [43]. Two cities in Norway achieved scale up across multiple sectors, including harm reduction programs, drop-in centers, shelters, prisons, medical facilities, and supervised injection facilities [44]. This Norwegian model included overdose education (OE) delivered to individuals and groups, and the study team provided an intranasal naloxone product at no cost to partner organizations. Centralized training of staff across 41 sessions with more than 500 staff resulted in the distribution of over 2000 units of naloxone over 18 months [45].

Taken together, the literature points to a promising array of implementation strategies that may increase OEND adoption and reach, but U.S. descriptions of OEND scale-up in diverse settings are limited. Furthermore, few studies have framed how implementation strategies may need to vary across phases of the scaling up effort, which the EPIS (Exploration, Preparation, Implementation, and Sustainment) framework suggests may need to occur [26].

This manuscript has two aims. Drawing upon the EPIS model [26] and inspired by efforts to expand medication for OUD through the “Hub and Spoke Model” [46], this paper describes a centralized “Naloxone Hub with Many Spokes” approach to implementing OEND in eight Kentucky communities as part of the HEALing (Helping to End Addiction Long-term^SM^) Communities Study (HCS). In addition to characterizing the Hub and Spoke Model with its implementation strategies deployed across the EPIS phases, this manuscript presents outcomes achieved by organizational partners, using the EPIS phases to characterize these implementation outcomes.

## Methods

### Study context

The HCS is a parallel-group, cluster randomized wait-list controlled trial testing the effects of the community-level Communities That HEAL (CTH) intervention on reducing opioid overdose deaths and a range of secondary, structural, and implementation outcomes in highly impacted communities in four states. Highly impacted communities within a state were defined as having a sum of ≥ 150 opioid-related overdose fatalities and a rate of ≥ 25 opioid-related overdose fatalities per 100,000 people in 2016; at least 30% of communities were required to be rural. Full details of the study protocol have been previously published [47]. In brief, the CTH seeks to engage and partner with opioid-focused community coalitions through a multi-phase process to promote the selection of EBPs within the Opioid-overdose Reduction Continuum of Care Approach (ORCCA) for implementation within the community [48–51]. OEND represents one of the three menus of EBPs within the ORCCA; the other two focus on medications for opioid use disorder (MOUD) and safer opioid prescribing and disposal. Given the variability in state contexts and community needs, the CTH intervention allows for considerable flexibility in the implementation strategies that research sites can employ to support the EBP implementation process.

Kentucky is one of four state-level sites in HCS with 16 counties serving as HCS communities (HCS-KY). These 9 urban counties and 7 rural counties represent more than 40% of the state population, and in 2019, had 42.3 opioid overdose deaths per 100,000 residents, with estimated rates of OUD that ranged from 2.9 to 12.2% among adults aged 18–64 [52]. Counties were categorized as rural or urban using the 2013 NCHS urban-rural classification scheme [53]. For more detail on characteristics of these Kentucky communities, see Drainoni et al. [54]. Eight HCS-KY counties were randomized to receive the CTH intervention as part of “Wave 1” (i.e., randomized to start the intervention first) which occurred from January 2020 through June 2022. Community coalitions set priorities about sectors and organizational venues for the implementation of OEND. Guided by those priorities, the HCS-KY research team developed implementation strategies to support OEND scale-up within organizational venues. Strategies were utilized at two levels: a centralized university-based naloxone dispensing unit (“Naloxone Hub”) and partner organizations (“Many Spokes”). This manuscript presents organizational data for eight Wave 1 communities in Kentucky from our interventional implementation study in which there was not a control group of organizations.

### Procedures: hub development and operations through the EPIS phases

The Naloxone Hub was located at the University of Kentucky in Lexington, KY. During the Exploration phase, implementation strategies for the Naloxone Hub included identifying funding sources to supplement the HCS-KY grant, identifying relevant state regulations, and soliciting input from regulatory experts on how to design a standing order agreement (SOA) for dispensing naloxone to partner organizations. HCS-KY funds were allocated to support faculty effort to design the OEND model as well as to purchase naloxone for the eight communities, but state stakeholders were also engaged to financially contribute to the purchasing of naloxone. State stakeholders agreed to provide over $885,000 from the Kentucky Opioid Response Effort’s SAMHSA funding for purchasing naloxone, which was added to funds allocated for naloxone purchasing through the HCS-KY grant.

States vary in terms of OEND-related policies, so consideration of the Kentucky regulatory environment was needed in the Exploration phase. Kentucky’s OEND regulations, most notably KRS 217.186, do not include a statewide standing order but do allow medical providers to utilize a standing order to prescribe and dispense naloxone to an agency for subsequent distribution to individuals [55]. KRS 217.186 requires education in overdose prevention, recognition, and response as part of naloxone distribution. Similar to many states, Kentucky state law provides immunity to the prescriber who has signed the standing order and immunity to persons who administer naloxone [56].

The Preparation phase involved hiring and training of Hub staff as well as designing standard operating procedures (SOPs), SOA templates, a data system to monitor spoke-level implementation, and materials to support partner organizations. Staffing included a full-time Naloxone Coordinator responsible for Hub operations and a team of seven Implementation Facilitators who were responsible for engaging with partner organizations to implement OEND and other EBPs selected by coalitions from the ORCCA. An SOP for Hub operations defined how naloxone nasal spray (Narcan®) would be ordered and secured, how it would be labeled and then transferred to agencies via trackable shipping or Hub staff, and how expiration dates would be monitored with procedures for retrieving expired units from partner organizations. An SOP for Implementation Facilitators explained the process of recruiting potential partner organizations, how to support partner organizations in designing an OEND workflow, and procedures for ongoing follow-up, naloxone inventory reconciliation, and technical assistance as needed. HCS-KY staff training focused on components of the SOPs with interactive practice used to develop facilitation skills.

Initially, a template for the SOA was written to describe the roles and responsibilities of partner organizations in delivering OEND so as to comply with Kentucky state law. This initial SOA, which was signed by an agency representative and HCS-KY’s addiction psychiatrist, authorized partner agencies to receive and store a supply of naloxone for their staff to distribute to individuals in the community. The SOA required agencies to provide overdose education that addressed seven key elements: (1) risk factors of opioid overdose; ​(2) strategies to prevent opioid overdose; (3) signs of opioid overdose; ​(4) steps in responding to an overdose; ​(5) information on naloxone; ​(6) procedures for administering naloxone; and ​(7) proper storage and expiration of naloxone. During the Implementation phase, some partner organizations that employed medical providers indicated a preference for relying on an SOA signed by their medical provider, so additional documents were drafted to support OEND via internal prescriptive authority. Agencies choosing to rely on internal prescriptive authority were instructed to develop a SOA (which could be based on the HCS-KY template), were provided a form to submit to the drug wholesaler so that HCS-KY-funded naloxone would be shipped to their agency (thus, bypassing the Hub), and were required to sign a memorandum of understanding between their agency and HCS-KY.

A data monitoring system was designed for inventory monitoring and collection of study-specific data. Using the Research Electronic Data Capture (REDCap) [57] application installed on computer tablets provided by HCS-KY, partner agencies were required to document distribution in an agency-specific REDCap project and asked to document the characteristics of OEND recipients in a second REDCap project. Although direct entry of data in real-time by the partner organization was preferred, paper surveys were provided if requested. Partner organizations were asked to sync their tablets to transmit these data to the Hub at least weekly.

Materials developed during the Preparation phase for partner organizations included OE materials as well as implementation support documents. Initial OE materials included an 10-min educational video and a trifold brochure, both of which focused on how to recognize an overdose, how to respond (i.e., administering intranasal naloxone, calling 911), and strategies for preventing opioid overdoses. The brochure included a QR code to the educational video which was available via YouTube and also loaded on computer tablets so that agencies could show the video without an Internet connection. The HCS-KY naloxone video and brochure were initially translated in Spanish but later, based on partner requests, were translated into Arabic, Swahili, and Kinyarwanda. In addition, partner organizations could use the interactive training developed by *Get Naloxone Now* (https://www.getnaloxonenow.org/#home), which met the requirements of the SOA. Two implementation documents were shared with partner organizations: a frequently asked questions (FAQ) document that provided key information about the HCS-KY naloxone program and a naloxone manual, which provided instructions on how to submit data via REDCap.

As the Hub moved into the Implementation phase, Hub staff engaged with community partner organizations by providing technical assistance, supplying agencies with naloxone, and ongoing expiration date monitoring. As the demand for naloxone increased over time, additional Naloxone Assistants were hired within the Hub to provide support to the Naloxone Coordinator and to provide additional technical support. The Naloxone Coordinator and Assistants met weekly with Prevention team faculty to discuss Hub operations throughout the Implementation phase. The team of Implementation Facilitators typically met at least twice weekly with the HCS-KY faculty and supervisors to discuss implementation progress and brainstorm ways to enhance implementation and troubleshoot challenges.

The Sustainment phase at the Hub-level focused on engagement with state-level stakeholders to transition partner organizations to a naloxone program supported by the Kentucky Opioid Response Effort (KORE) through SAMSHA funding and operated by the Kentucky Pharmacists Association (KPhA). Meetings were held with key state stakeholders to ensure funding for additional naloxone that partner organizations could order from the KORE-KPhA program via a web portal.

### Procedures: partner organizations and the EPIS model

The EPIS model also guided work with the “many spokes,” which represented organizations in the sectors of health care, behavioral health, and criminal justice that were recruited to partner with HCS-KY in OEND implementation. Coalitions prioritized organizational venues for HCS-KY OEND partnerships, informed by a landscape analysis that identified organizations within the prioritized venues; over the CTH Intervention period, if additional organizations were identified through community contacts that aligned with the coalitions’ prioritized venues, those organizations were added to the Hub’s workflow. The implementation process with the “many spokes” relied heavily on a team of Implementation Facilitators employed at the Hub who worked with organizations throughout the EPIS phases.

During Exploration, Implementation Facilitators engaged and recruited community partner organizations from prioritized venues. In some cases, community coordinators employed by HCS-KY and coalition members were able to make warm handoffs to introduce Implementation Facilitators to potential partner organizations. In many cases, initial contact represented “cold calling” via an email template and telephone follow-up to non-responsive agencies. This template briefly summarized the study’s goals and the resources that HCS-KY could provide to agencies interested in joining the OEND program.

Agencies indicating interest were then scheduled for an initial meeting with an Implementation Facilitator. Implementation Facilitators worked to move interested organizations quickly into the Preparation phase through facilitating an initial meeting that provided information about the HCS-KY Hub, described options for overdose education that would meet the SOA requirements, sought to identify an OEND liaison, and designed an OEND workflow that would meet the needs of the partner organization. If needed, additional meetings were facilitated until the OEND workflow was developed. Implementation Facilitators were responsible for obtaining the SOA that listed all personnel who would be involved in OEND and was signed by the agency; the signed SOA was then transferred to the Naloxone Coordinator who ensured the SOA was signed by the HCS-KY physician. Once the SOA was fully executed, the Implementation Facilitator scheduled a meeting between the partner organization and the Naloxone Coordinator for a training on the data requirements and how to use the REDCap system and provided tablets for documenting distribution and recipient demographics.

During Implementation, agency staff delivered OEND to clients while Implementation Facilitators engaged in ongoing technical assistance with community partner agencies, and the Naloxone Coordinator ensured agencies had sufficient naloxone. Implementation Facilitators held an initial follow-up meeting 1-month after partner organizations received their first shipment of naloxone. In cases where implementation was well underway and weekly data submissions were being received, partner organizations were subsequently scheduled for quarterly follow-up meetings, but Implementation Facilitators used their discretion to schedule more frequent meetings to troubleshoot challenges. A follow-up meeting guide was utilized as a framework for discussions around successes and challenges, to reconcile naloxone inventory with data submitted regarding distribution, and to make updates to staff listed on the SOA. In situations where agencies experienced significant staff turnover, Implementation Facilitators worked to train new staff about the HCS-KY OEND program.

Finally, Implementation Facilitators worked to support the transition of partner organizations to the state’s KORE-KPhA naloxone program during the Sustainment phase (July 2022–December 2022). Implementation Facilitators held a final meeting with partner organizations to discuss strengths and weaknesses of the agency’s participation in the HCS-KY OEND program, to share information about how to enroll in the KORE-KPhA naloxone program, and to determine if there was a need for a final naloxone shipment to serve as a bridge until the agency transitioned to the KORE-KPhA naloxone program. Until that final meeting, Implementation Facilitators held follow-up meetings, as needed, to reconcile naloxone inventory and work with agencies on replenishment shipments.

### Impact of the COVID-19 pandemic

The CTH intervention was launched in January 2020, just 3 months before the COVID-19 pandemic was declared a national emergency. Early reports of COVID-19 disrupting opioid-related services [58], including operation of SSPs [59], as well as mass releases of individuals incarcerated in jails [60], prompted a change to the CTH intervention to allow for OEND scale-up to begin in jails, SSPs, and addiction treatment and recovery facilities, if approved by the HCS coalitions. Initiation of this “fast-track OEND” effort occurred about 6–8 months before coalitions had completed the broader EBP selection process, and the HCS-KY team greatly accelerated its effort to move through the EPIS phases to launch the Naloxone Hub and begin recruiting organizational partners.

Another significant change was the shift to remote work for the Implementation Facilitators. The original facilitation model was designed around in-person initial meetings with potential partner organizations, but then shifting to video conferencing for follow-up meetings. The COVID-19 pandemic meant that video conferencing (i.e., Zoom®) was used throughout Wave 1. It was only in mid-2022 that Implementation Facilitators occasionally held in-person follow-up meetings with partner organizations.

Finally, the COVID-19 pandemic eliminated in-person services in some settings, which resulted in additional OEND models. Most notable was OEND in the eight county-level probation and parole offices and the services offered by the Department of Pretrial Services in the eight counties, where the lack of in-person services required modification to the OEND model. Clients of these partnering agencies could request naloxone directly from the Naloxone Hub after watching the HCS-KY video on a study-funded website; when in-person services resumed, HCS-KY-funded staff were deployed to parole/probation offices to deliver OEND. However, OEND remained available only by self-request via the website for pretrial services clients. These implementation efforts sufficiently deviated from the main HCS-KY model whereby partner agency staff delivered OEND that implementation outcomes for probation and parole and pretrial services are not reported in this manuscript.

### Data collection, measures, and analysis

Data on implementation outcomes were drawn from two sources: (a) internal study management systems where OEND implementation progress was tracked over time by the Naloxone Coordinator and Implementation Facilitators, and (b) REDCap data submissions on naloxone distribution from organizational partners. As potential partner organizations were identified, they were added to a tracker maintained by the Implementation Facilitators that was updated as organizations agreed to initial meetings, agreed to partner, and returned the signed SOA. Once an organization submitted a signed SOA, the Naloxone Coordinator set up the partner-specific REDCap project for tracking distribution.

Implementation outcomes were measured across the EPIS phases. During the Exploration/Preparation phase, the outcome was the percentage of agencies that were contacted and agreed to an initial meeting to learn about the HCS-KY naloxone program and begin designing an OEND workflow. Adoption of the HCS-KY naloxone program was measured during the Implementation phase and represented the percentage of contacted agencies who agreed to implement OEND and received at least one shipment of naloxone during the Wave 1 intervention period (January 2020–June 2022). Adopting organizations were coded for organizational type for descriptive purposes. Reach was measured during the Implementation phase of the Wave 1 intervention and consisted of: (1) the total number of naloxone units (i.e., package containing two doses of intranasal naloxone) distributed by partner agencies, (2) the mean total number of units distributed per agency, and (3) a categorical measure of total distribution during the Implementation phase that divided agencies into groups: 0, 1–20, 21–100, 101–300, 301–1000, and > 1000 units distributed. These data on reach were submitted by partner agencies via REDCap to the Hub; during follow-up meetings, Implementation Facilitators shared information on total distributions received via REDCap and asked agencies to count remaining units on hand in order to reconcile distribution counts relative to units dispensed by the Hub, thus eliminating missing data. The mean number of units distributed per month during the agency’s specific period of implementation was also calculated, as potential partners were recruited on a rolling basis during the Implementation phase. A small number of agencies (n = 13) requested naloxone to have on-site in order to respond to overdoses at their facilities; these units dispensed for on-site administration were not included in the measures of reach. Given that units of naloxone represent count data, negative binomial regression [61] was used to examine whether the total distributed units was associated with organizational type (with MOUD agencies as the reference category) and rurality (1 = rural, 0 = urban), while controlling for months of implementation. The number of units distributed by agency partners during the Implementation phase were also aggregated to the community-level to measure the mean number of units and a population-adjusted measure of naloxone saturation (i.e., number of units per 100,000 residents) using 2021 population estimates [62]. For the Sustainment phase (July 2022–December 2022), the outcome measured was the percentage of adopting agencies that attended a sustainment meeting and agreed to transition to the KORE-KPhA naloxone program as well as monthly naloxone reach (i.e., number of distributed units divided by 6 months). A paired *t*-test was used to compare monthly naloxone reach during the Implementation phase to monthly naloxone reach during the Sustainment phase. Data analyses consisting of descriptive statistics for these implementation outcomes were pre-planned, but the negative binomial regression model and the paired *t*-test to compare monthly reach during the Implementation and Sustainment phases were post-hoc analyses. All analyses were conducted using Stata 17.0 (StataCorp, College Station, TX).

## Results

Over the course of the Wave 1 intervention period, 209 organizations in eight counties were identified as potential partners for OEND implementation and contacted by an Implementation Facilitator. About 84.7% (n = 177) of these organizations engaged in the Exploration/Preparation phase by participating in an initial meeting with an Implementation Facilitator about the HCS-KY OEND program. Those that did not participate in the Exploration/Preparation phase consisted of organizations that were unresponsive to multiple contacts, those that declined due to lack of interest, and those that already had implemented OEND.

Adoption of the HCS-KY OEND program, which was defined as the agency receiving at least one shipment of naloxone, was achieved with 69.4% (n = 145) of all organizations contacted and 81.4% of the organizations that attended an initial meeting with an Implementation Facilitator. Agencies that engaged in the Exploration/Preparation phase but did not adopt OEND typically cited insufficient staffing as a barrier to adoption. As seen in Table 1, the most common types of organizational settings were MOUD clinics, non-MOUD addiction treatment and recovery service organizations, and outpatient medical clinics.

**Table 1:** Distribution of HCS-KY OEND adopters (n = 145) by organizational type

Organizational type	% (N)
Medication for opioid use disorder (MOUD) clinics	30.3% (44)
Non-MOUD addiction treatment and recovery services	19.3% (28)
Outpatient medical clinics (i.e., non-hospital/non-MOUD)	16.6% (24)
Social services (e.g., Department of Community-Based Services, homeless shelters)	10.3% (15)
Drug courts and private alternatives to incarceration	5.5% (8)
Health departments and syringe service programs embedded in health departments	5.5% (8)
Jails	4.8% (7)
Emergency response (ambulance or fire)	4.1% (6)
Hospitals	2.1% (3)
Dental clinics	1.4% (2)

Percentages do not sum to 100% due to rounding

**Fig. 1:**
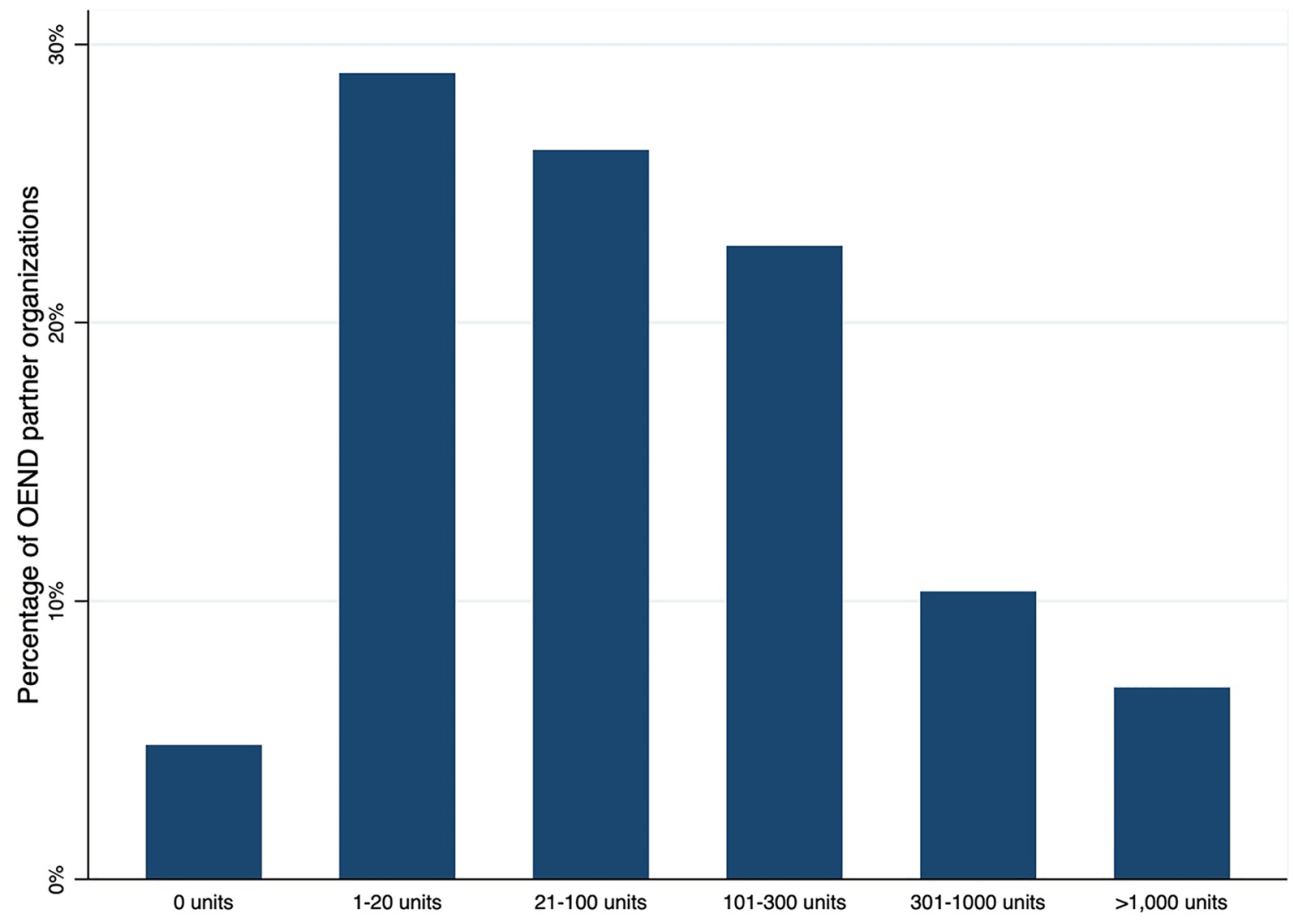
Percentage of HCS-KY OEND partner organizations across reach categories

The first distributions of HCS-KY-funded naloxone occurred in April 2020, just 4 months after the start of the CTH intervention in Wave 1 communities. In terms of overall reach during the Wave 1 intervention period, 145 HCS-KY OEND partner organizations distributed a total of 40,822 units of naloxone. Figure 1 categorizes the reach achieved by partner organizations. About 55.2% (n = 80) of adopting agencies distributed 100 units or less, while 17.2% distributed more than 300 units. A small number of partner organizations (4.8%; n = 7) received a shipment of naloxone but were not successful in distributing any units of naloxone during the Wave 1 intervention period.

Partner organizations distributed a mean of 281.5 units of naloxone (SD = 806.2). Reach per month was also calculated to account for the varying amount of time that partner agencies engaged with HCS-KY; on average, agencies distributed 14.8 units per month (SD = 34.6; range = 0.0–296.4). A negative binomial regression model estimated associations between organizational type, rurality, and months of implementation on naloxone reach (Table 2). Controlling for organizational type and months of implementation, rurality was not significantly associated with naloxone reach. There were two significant differences by organizational type. Healthcare organizations had significantly less naloxone reach than MOUD agencies, while health departments had significantly greater naloxone reach than MOUD agencies. Months of implementation was positively correlated with the reach categories.

**Table 2:** Negative binomial regression model of naloxone reach

Variable	Incidence rate ratio coefficient (95% CI)	*p*-value
Rural location (vs. urban location)	0.82 (0.50, 1.36)	0.44
Organizational Type		
Medication for opioid use disorder (MOUD) clinics	Reference	
Non-MOUD addiction treatment and recovery services	1.41 (0.76, 2.61)	0.27
Healthcare organizations	0.43 (0.20, 0.90)	0.03
Social services (e.g., Department of Community-Based Services, homeless shelters)	0.83 (0.37, 1.85)	0.64
Jails	2.13 (0.77, 5.89)	0.14
Drug courts and private alternatives to incarceration	0.57 (0.21, 1.56)	0.27
Emergency response (ambulance or fire)	0.60 (0.20, 1.80)	0.37
Health departments and syringe service programs embedded in health departments	4.52 (1.70, 12.04)	0.003
Months of implementation	1.15 (1.11, 1.20)	< 0.001
*Constant*	17.42 (7.43, 40.85)	
*Alpha*	1.61 (1.31, 1.99)	

The likelihood ratio test for the alpha statistic was significant (p < 0.001), indicating that negative binomial regression was more appropriate than Poisson regression. Healthcare organizations included outpatient clinics not providing MOUD, hospitals, and dental clinics; these dental clinics were owned by a healthcare organization that largely provides primary care

In terms of naloxone reach at the county level over the Implementation phase, the mean number of units distributed was 5102.8 (SD = 3653.3; range = 1057 − 11,053). The mean county level distribution rate was 8396.5 units per 100,000 residents (SD = 8103.1; range = 1709.5–25,296.3). County-level total distribution and distribution rates per 100,000 residents achieved by HCS partner agencies are presented in Table 3.

**Table 3:** County-level naloxone distribution by HCS partner agencies

County	Rural or urban status	Total units	Units per 100,000 residents
County A	Rural	11,053	11,675.8
County B	Urban	9327	25,296.3
County C	Urban	6029	12,586.9
County D	Urban	5501	1709.5
County E	Urban	4141	2443.1
County F	Rural	2488	8091.8
County G	Rural	1057	2996.5
County H	Rural	1226	2372.2

After the Wave 1 intervention period, the HCS-KY OEND program continued to provide naloxone and implementation facilitation until plans could be developed with partner organizations to transition them to the KORE-KPhA naloxone program, with all support ending by December 2022 at the latest. Of the partner organizations that adopted the HCS-KY OEND program, 87.6% (n = 127) attended a sustainability meeting with an Implementation Facilitator and agreed to transition to the KORE-KPhA naloxone program. During this transition period and conservatively using 6 months as the denominator, partner organizations distributed a mean of 7.7 HCS-funded naloxone units per month, which is a significant decrease from the monthly mean of 14.8 units per month during the Implementation phase (*t =* 3.11, *df* = 144, *p* = 0.002). This rate of distribution may be somewhat conservative in that agencies may have started to receive naloxone from KORE-KPhA before the end of this 6-month period.

## Discussion

The “Hub with Many Spokes” model of OEND scale-up was feasible in eight Kentucky counties, with greatly expanded naloxone distribution over the Implementation phase by 145 partner agencies that represented a diverse array of organizational types. This degree of scale-up was achieved through an approach that used the EPIS Model to organize a set of implementation strategies, that resulting in more than 40,000 units of naloxone being distributed by these partners to individual community members.

To contextualize the potential impact of this level of reach, it is useful to consider modeling studies that have estimated how many units of naloxone are needed to impact opioid overdose deaths. Canadian modeling data suggests that distribution of 11 units is needed to avert one death [16]; by that metric, the reach that HCS-KY OEND would have the potential to avert about 3700 deaths. Using a 5-year time horizon, U.S. modeling that accounts for the rise in fentanyl and the impact of the COVID-19 pandemic indicates an 5% expansion of OEND would result in a 4% decrease in deaths, while a 30% expansion would decrease deaths by 26% [17]. Recent modeling research has produced state-specific estimates of how varying levels of naloxone distribution through different models of OEND would impact overdose deaths [19]. For the Kentucky estimates presented in Irvine et al., community distribution, which is the model described here, had nearly double the impact of pharmacy-initiated naloxone while expanding naloxone by prescription was likely to have much lower impact. The Kentucky-specific community distribution estimates published by Irvine and colleagues indicated that naloxone distribution rates of 1000 units per 100,000 population would reduce the opioid overdose mortality rate by 6.6 deaths per 100,000. Our overall distribution rate was more than 5000 units per 100,000 population, which presumably should substantially reduce the opioid overdose mortality rates of these eight counties over time; the impact on opioid overdose mortality will be the focus of future manuscripts. However, it should be noted that scaling up additional interventions, such as MOUD, are important to achieve large-scale reductions in overdose deaths [63].

In considering factors associated with scaling up OEND, there was no association for rurality but some differences by organizational type. In selecting counties, our team used several criteria for inclusion, including the presence of a syringe service program, a jail, and at least one buprenorphine-waivered provider [47]. These criteria meant that rural counties had at least some degree of harm reduction and treatment infrastructure, which may have reduced some rural-urban differences that would have occurred if the rural areas had lacked such resources. Regarding organizational type, healthcare organizations achieved less naloxone distribution than MOUD agencies, likely reflecting the impact of the COVID-19 pandemic which was causing substantial morbidity and mortality during the intervention period. The urgent demands of treating individuals with COVID and other competing demands (e.g., rollout of the vaccines) likely made it difficult for healthcare organizations to dedicate sufficient staff time to implementing OEND. Health departments, which operated all of the SSPs in these eight counties, also were impacted by the COVID-19 pandemic, but likely recognized the increased urgency of scaling up OEND as overdose deaths increased in the early months of the pandemic [3]. SSP staff were innovative in terms of moving SSP operations outdoors in response to the COVID-19 pandemic, while the larger health departments also partnered to implement web-based overdose education with mail-based naloxone distribution. Two SSPs distributed large quantities of naloxone, such that they represented 41% and 67% of all units within those counties. Overall, much of the naloxone distributed by partner agencies went to individuals at high risk of opioid overdose or individuals with at-risk individuals in their social networks. Anonymous survey data was received by the Hub on individual-level characteristics for about two-thirds of units distributed, and of these, 69% of units went to a person who had previously witnessed an overdose and 41% went to a person who reported a personal history of overdose [64].

Experiences with scaling up OEND in these eight counties led to some adaptations during the Implementation phase and subsequent refinements for the second wave of communities; the refined materials related to the implementation process are available for download (see Additional file 1). The urgent need to scale-up OEND in the context of the COVID-19 pandemic meant there was not time to use the implementation strategy of in-depth needs assessments prior to designing the HCS-KY OEND model, so we instead worked to incorporate partner feedback obtained by the Implementation Facilitators as quickly as possible. Feedback from some early partner organizations prompted the translation of the overdose education materials into more languages and development of the internal prescriptive authority option. We initially had planned on the HCS-KY video being the primary method for overdose education, but when some partners indicated that showing the video on the study-supplied tablet would still be challenging in terms of workflow, we adapted the OE model to include verbal review of the HCS-KY naloxone brochure.

Even with study-supplied computer tablets, there were technological challenges for some agencies in providing distribution data. We decided to use the mobile application feature of REDCap because it allowed data to be stored on the tablets; we believed it would be helpful for agencies that may be delivering OEND in contexts where Wi-Fi was not readily available. The REDCap distribution project was designed so that agencies should scan a QR code on each unit of naloxone as it was being distributed. However, many agencies struggled with scanning the QR codes and uploading data to the Naloxone Hub, and Implementation Facilitators spent considerable time providing technical assistance around data submission. For Wave 2 communities, we are not using the REDCap mobile application; instead, each agency has a unique online survey link to document OEND without using a QR code, which should diminish many of the challenges associated with obtaining distribution data.

This study was conducted when naloxone was only available as a prescription medication, which is why the standing order agreements between HCS-KY and the agencies was a key element of the model. On March 29, 2023, the U.S. Food and Drug Administration announced its approval of Narcan® nasal spray for over-the-counter (OTC) status [65], a regulatory change sought by advocates, health care providers, and researchers [66, 67]. OTC naloxone should improve access to this lifesaving medication [68], although there are concerns about whether insurance companies will still include OTC naloxone on their formularies [69]. Stigma remains entrenched in many communities, which may still prevent some individuals from purchasing naloxone. Pricing may place OTC naloxone out of reach of some individuals. Thus, even with OTC status, we anticipate that community distribution will still play an important role in scaling up OEND, with the added benefit of overdose education being provided to improve skills at recognizing and responding to an overdose. Furthermore, the “Hub and Many Spokes” model offers valuable technical assistance and coordination to integrate OEND into the usual workflow of community partner agencies.

Several limitations should be noted. First, these findings represent implementation efforts in eight counties in a single state. The counties were a mix of urban and rural communities, but states vary considerably in both policies and financial resources that are relevant for OEND implementation. Our findings also cannot speak to the optimal “dose” of implementation facilitation because our SOP allowed for considerable discretion by the Implementation Facilitators in terms of tailoring the scheduling of follow-up meetings with agencies. From our perspective, the urgency of scaling up OEND outweighed considerations around limiting how much facilitation that the team was willing to provide. Finally, we are in the process of conducting qualitative interviews with organizational partners, and we anticipate the qualitative data will yield rich insights into the implementation process and prospects for longer term sustainment.

## Conclusion

Given the magnitude of the opioid epidemic, efforts to scale up evidence-based practices, such as OEND, are critically important to saving lives and mitigating the harms of OUD. Implementation science frameworks and implementation strategies can inform approaches to scaling up OEND, which should improve public health over time. As demonstrated by our work in eight Kentucky communities, OEND scale-up was achievable using a “Hub with Many Spokes” model to increase access to naloxone.

### Supplementary Information

**Additional file 1.** Implementation Scale-Up Materials by EPIS Phase.

## Data Availability

Implementation-related materials are provided in Additional file 1. Data reported in the current study are not publicly available to protect the privacy of organizational partners.

## Abbreviations

CTH: Communities That HEAL
EBP: Evidence-based practice
EPIS: Exploration, preparation, implementation, and sustainment
FAQ: Frequently asked questions
HCS: HEALing (Helping to End Addiction Long-term^SM^) Communities Study
HCS-KY: HEALing Communities Study Kentucky site
KORE: Kentucky Opioid Response Effort
KPhA: Kentucky Pharmacists Association
MOUD: Medication for opioid use disorder
SSPs: Syringe service programs
OE: Overdose education
OEND: Overdose education and naloxone distribution
ORCCA: Opioid-overdose Reduction Continuum of Care Approach
OTC: Over-the-counter
OUD: Opioid use disorder
REDCap: Research Electronic Data Capture
SAMSHA: Substance Abuse and Mental Health Services Administration
SOA: Standing order agreement
SOP: Standard operating procedure
US: United States
