# Supplementary material for: Scaling up overdose education and naloxone distribution in Kentucky: adoption and reach achieved through a “hub with many spokes” model
Source: Addict Sci Clin Pract. 2023 Nov 30;18:72. doi: 10.1186/s13722-023-00426-6 (PMC10688494; doi:10.1186/s13722-023-00426-6)
Supplement: Supplementary file 1 — Additional file 1. Implementation Scale-Up Materials by EPIS Phase. [file 13722_2023_426_MOESM1_ESM.pdf]

Implementation Scale-Up Materials by EPIS Phase

Scaling Up Overdose Education and Naloxone Distribution in Kentucky: A Hub with Many Spokes Model

Additional File 1

Hannah K. Knudsen, Patricia Freeman, Doug Oyler, Carrie Oser, and Sharon L. Walsh

University of Kentucky

Table of Contents

Exploration Phase: Materials Emailed to Agency before Initial Meeting
HCS-KY Naloxone Flyer……………..……………..……………..……………………..3
OEND Models Flyer……………………..……………………..……………………………..4

Preparation Phase: Implementation Facilitation Materials for Initial Meeting and Implementation Planning
Opportunities for Expanding Overdose Education & Naloxone Distribution Slides…...…6
Initial Meeting Guide………………...……………………………………………………...14
Standing Order Agreement……………...……………………………………………………18

Implementation Phase: Implementation Supports and Implementation Facilitation Materials for Follow-up Meetings
Overdose Education: HCS-KY Naloxone Video YouTube Links….……………………..20
Overdose Education: HCS-KY Naloxone Brochure in English, Spanish, and Arabic.…21
HCS-KY OEND Frequently Asked Questions (FAQ)……….………………………………..27
Naloxone Procedures for HCS-KY Partner Agencies………………………….……….…33
Data Collection: Naloxone Training and Distribution Form……...………………………..37
Follow-up Meeting Guide……………...…………………………………………………...38
Naloxone Report for Partner Agencies………...……………………………………………42

Sustainability Phase: Implementation Facilitation Materials for Sustainability Meeting
Sustainability Meeting Guide………...…………………………………………………43
OEND Summary Report………...………………………………………………………49

HCS Naloxone Progam

A Partnership to Save Lives

NIH HEAL INITIATIVE

HEALing Communities Study Kentucky

The HEALing Communities Study is partnering with your community to establish and expand overdose education and naloxone distribution.

Providing naloxone and overdose training reduces opioid overdose deaths and improves outcomes for individuals, families, and communities.

The HCS Naloxone Program is eager to assist your organization and help protect the at-risk individuals you serve.

How the Program Works

HCS		Agency
Assists with planning	→ ←	Identifies key staff roles
Signs standing order agreement	→ ←	Signs standing order agreement
Purchases & dispenses naloxone	→ →	Receives naloxone
Supports naloxone distribution through ongoing training, materials, and technical assistance	→	
	←	Trains individuals in overdose preventionand response
	←	Distributes naloxone
Reviews reports & replenishes naloxone supply	← ←	Documents and reports training and distribution
	→	

No-Cost Resources Available from HCS

- Narcan Nasal Spray
- Online overdose education
- Train-the-trainer overdose education sessions for staff
- Materials and technology to support program
- Ongoing technical assistance

The HEALing Communities Study

HCS is an $87 million project funded by the National Institute on Drug Abuse with a goal of reducing opioid overdose deaths by 40 percent in 3 years.

Researchers from the University of Kentucky will work with coalitions in 16 Kentucky counties to leverage existing resources and develop a collaborative model for ending the opioid overdose crisis.

The intervention strategies focus on expanding treatment for opioid use disorder, ensuring naloxone availability, and improving prescription opioid safety.

NIH
HEAL
INITIATIVE

HEALing Communities Study
Kentucky

Overdose Education and Naloxone Distribution Models

AVAILABLE OPTIONS FOR OVERDOSE EDUCATION

Audiovisual

HEALing Communities Study Video

This 9-minute video reviews opioid overdose prevention, recognition and response, including how to administer intranasal naloxone. It also provides brief guidance on additional steps to take during the COVID-19 pandemic. It includes voice over PowerPoint slides, as well as demonstration of key elements by trained actors.

How can videos be used?

In-Person Overdose Education

- Video is loaded on a tablet or other device for use with an individual client at the agency.
- Video is loaded on a computer or smart TV for use with a group of clients at the agency.

Virtual Overdose Education

- Video is accessible online for individuals to complete on their own at a time and location convenient for them.
- Video is shown during a virtual training held online at a scheduled time via video conferencing (i.e., a webinar).

Written

HEALing Communities Pamphlet

The HEALing Communities educational pamphlets are brochures to review with clients focused on key elements of overdose prevention, recognition, and response. Pamphlets are available in English, Spanish, and Arabic language versions.

How can pamphlets be used?

In-Person Overdose Education

- Face-to-face training is delivered by agency personnel in which the pamphlet is reviewed.

Virtual Overdose Education

- Pamphlets may be used as a handout for take-home review to support other types of overdose education (e.g., audiovisual).

NOTE: All naloxone sent by HEALing Communities study is shipped with the educational pamphlet.

NIH HEAL INITIATIVE

HEALing Communities Study Kentucky

Overdose Education and Naloxone Distribution Models

AVAILABLE OPTIONS FOR NALOXONE DISTRIBUTION

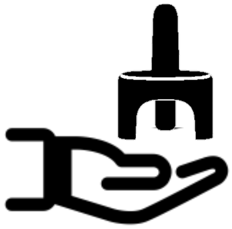

In-Person Distribution

Your agency receives and stores naloxone inventory from the HEALing Communities Study to be distributed by agency personnel to individuals on-site at the agency or in the community. Prior to distributing naloxone, agencies must confirm that overdose education has been provided to each individual.

How are education and distribution documented?

Online

- Your agency completes a brief survey online using the REDCap survey tool.
- Your can also complete a paper survey and enter information online within 1 week of distribution.

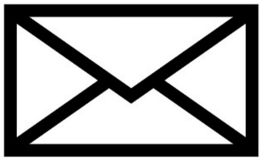

Mail Distribution

Your agency documents overdose education via approved on-demand or virtual training and distributes naloxone via mail to documented learners.

How are education and distribution documented?

- HEALing Communities Study generates an online Typeform account for your agency allowing clients to complete education and enter demographics.
- Your agency downloads responses for clients and sends to the study team.

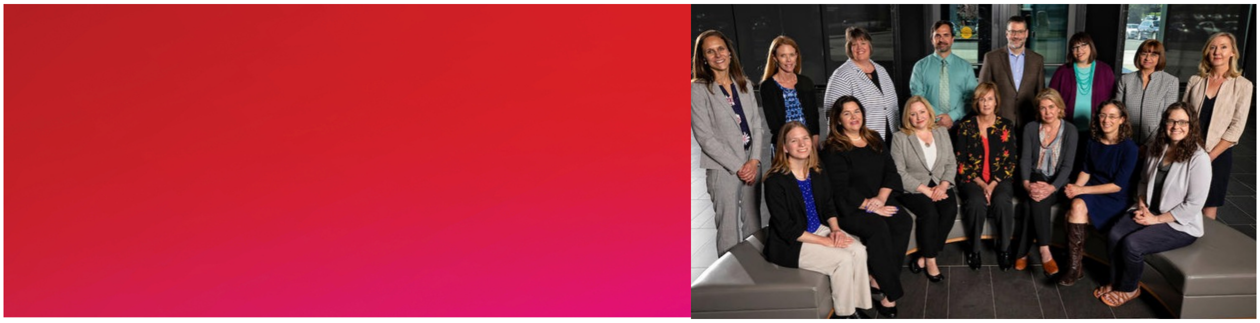

NIH
HEAL
INITIATIVE
HEALing Communities Study
Kentucky

Opportunities for Expanding Overdose Education & Naloxone Distribution

Sharon Walsh, Principal Investigator, sharon.walsh@uky.edu
Implementation Facilitator, Name, Email

University of Kentucky | Boston Medical Center | Columbia University in the City of New York | The Ohio State University | RTI International | SAMHSA Substance Abuse and Mental Health Services Administration | NIH National Institute on Drug Abuse

NIH HEAL Initiative and Helping to End Addiction Long-term are service marks of the U.S. Department of Health and Human Services.

Today's Agenda

- Provide a brief overview of the HEALing Communities Study
- Discuss potential partnership to expand overdose education and naloxone distribution

Overview of the HEALing Communities Study

- A partnership with the National Institutes of Health (NIH) and the Substance Abuse and Mental Health Services Administration (SAMHSA)

- Our project is being conducted in partnership with numerous federal, state, community, behavioral health, health care, public health, and criminal justice partners
  - Secretary of the Cabinet for Health & Family Services
  - Secretary of the Justice & Public Safety Cabinet

- $87 million was awarded to the University of Kentucky

Study Design & Goal

- To test the effectiveness of a community-engaged intervention designed to increase the use of evidence-based practices to reduce opioid overdose deaths
  - Working with local community coalitions to set priorities for how to reduce overdose deaths
- Goal is to reduce opioid overdose deaths by:
  - Increasing overdose education and distribution of naloxone (Narcan®)
  - Increasing the number of individuals receiving medication treatment
  - Increasing long-term treatment retention & recovery support services
  - Reducing high-risk opioid prescribing

NIH HEAL INITIATIVE
HEALing Communities Study Kentucky

Naloxone

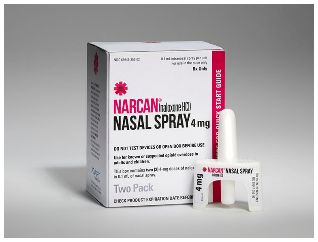

- Reverses an opioid overdose
- Naloxone is safe
  - No potential for misuse & no interactions with other medications
  - Causes no harm if used by mistake
- Research indicates:
  - Lack of sufficient training in administering naloxone is a barrier to effectively using it
  - Single greatest predictor of successfully reversing an overdose is carrying naloxone

The HEALing Communities Study wants to help!

- HCS can provide resources at no-cost
  - Overdose education videos
  - Tablet(s) for use during overdose education
  - Naloxone (Narcan® nasal spray)
- Our goal today is to discuss a partnership

NIH HEAL INITIATIVE
HEALing Communities Study Kentucky

NIH HEAL INITIATIVE

HEALing Communities Study Kentucky

HCS Naloxone Program

University of Kentucky · Boston Medical Center · Columbia University in the City of New York · The Ohio State University · RTI International · SAMHSA · NIH National Institute on Drug Abuse

NIH HEAL Initiative and Helping to End Addiction Long-term are service marks of the U.S. Department of Health and Human Services.

HCS Naloxone Program

- HCS physician dispenses naloxone to partner agencies under standing order agreement
- Partner agencies distribute naloxone to individuals who have been trained in overdose prevention and response
- No need for patient name on naloxone distributed
- Partner agencies provide documentation of naloxone distribution
  - Very short anonymous survey

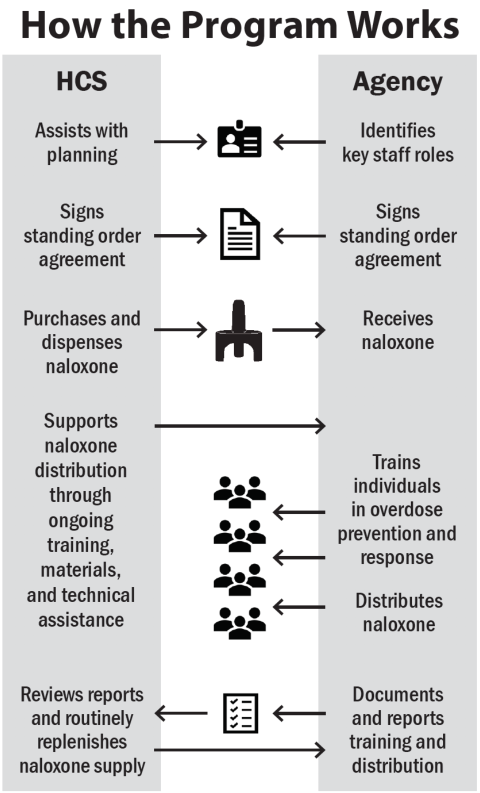

How Are We Able to Do This? The HCS Standing Order Agreement

- Standing order agreements are made possible by Kentucky state law (KRS 217.186)
  - Our SOA has been approved by Mike Rodman, executive director of Kentucky Board of Medical Licensure
- Serves as "prescription," authorizing dispensing of Narcan Nasal Spray from HCS to partner agency
- Authorizes partner agency to maintain supply and distribute naloxone to any individual who has completed bystander training in overdose prevention, recognition, and response
- Signed by Dr. Michelle Lofwall and an agency representative
  - Contains list of all staff who will be involved in OEND

NIH HEAL INITIATIVE
HEALing Communities Study Kentucky

Required Overdose Education by Agency Staff when Distributing Naloxone under the SOA

- Overdose education must cover these elements:
  - (1) Risk factors of opioid overdose;
  - (2) Strategies to prevent opioid overdose;
  - (3) Signs of opioid overdose;
  - (4) Steps in responding to an overdose;
  - (5) Information on naloxone;
  - (6) Procedures for administering naloxone; and
  - (7) Proper storage and expiration of naloxone product
- Can use HCS video or in-person training by a competent individual (e.g., verbally review HCS brochure)

Documenting Naloxone Distribution

- After providing overdose education, agency staff documents naloxone distribution
- Anonymous web survey
  - Two questions to document OEND
  - Short demographic questions
- Two reasons documentation is important
  - HCS uses this data to identify when to send another shipment of naloxone
  - We must report counts of naloxone units and demographics as part of the study

How to Document Every Unit of HCS Naloxone

Video about documenting via REDCap shown during meeting is posted at: https://www.youtube.com/watch?v=bCJzjYUuHz8.

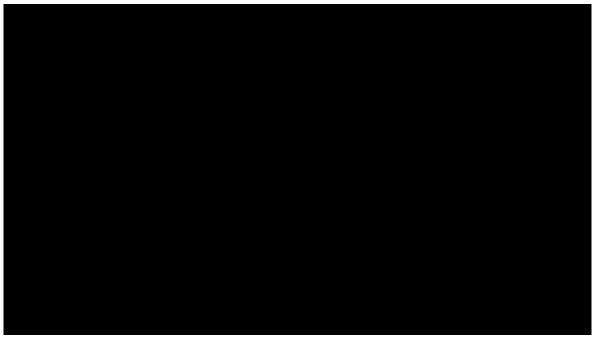

Next Steps

Next Steps

- Identify an OEND Liaison
  - Tracks completion of overdose education
  - Responsible for tracking distribution of naloxone
- We will send the standing order agreement and our FAQ
  - After review, the SOA requires:
    - Signature of agency representative
    - Names of all individuals who will be distributing naloxone
- Once we receive the SOA, HCS Naloxone Program Manager will send OEND supplies
- Periodic follow-up meetings after OEND starts

KY-OPEN
Overdose Prevention Education Network

CE available for physicians, nurses, pharmacists, social workers, and KY Licensed Drug & Alcohol Counselors

Scan for live sessions

https://bit.ly/kyopen-signup

Scan for online sessions

http://Cecentral.com/kyopen

- Continuing education with OUD clinical experts
  - Open to all professions
    - Weekly sessions via Zoom, Thursdays 11:30 AM – 1PM
    - Online accredited enduring sessions available: http://cecentral.com/kyopen
  - 1st week is didactic on the topic
  - 2nd week is Q&A and case discussions on the topic

KY-OPEN
Overdose Prevention Education Network

- Join the KY-OPEN Weekly Newsletter
  - Registration for our weekly live sessions with CME
  - Q&As with clinical experts
  - Community Spotlights
  - Links to resources and events throughout Kentucky
- To Register
  - Visit https://bit.ly/kyopen-signup
  - Email HCSEducation@uky.edu

- CME available for physicians, nurses, pharmacists, social workers, and KY Licensed Drug & Alcohol Counselors
- Online accredited enduring sessions available:
  - Visit http://cecenteral.com/ky-open

Scan for newsletter

Scan for online sessions

Overdose Education and Naloxone Distribution-Standing Order Agreement Model

INITIAL MEETING GUIDE
Wave 2

Before the Meeting

- Add your name and email address to Slide 1 in PowerPoint file
- Prepare first draft of Partner Organizational Planning Tool (POPT)
- Send Outlook/Zoom invitation with the PowerPoint file and other materials
- Be ready to screen-share the PowerPoint file during the meeting

Meeting Date: _______________

Meeting Start Time: __________

Meeting End Time: ___________

TASK: *During the introductions in the Zoom meeting, document the names and titles of individuals participating in the meeting.*

TASK: *Provide Overview of HCS via Slides 1-6 and set the agenda for today's meeting using the Introduction to HCS OEND-SOA PowerPoint. Be sure to tailor your presentation of the Overview to HCS to the audience:*

- *If the meeting is with a single representative from the agency who has been participating in the HCS-designated coalition, try to frame the initial overview slides as "a reminder" and move quickly through the overview slides.*
- *The same applies if multiple people are present and they have all been involved in the coalition.*
- *If multiple people are present for the meeting but not all of them have been involved in the coalition, acknowledge that some may have heard more about the study, but these few slides are just to make sure everyone is on the same page.*

After presenting slides 1-6, stop the presentation.

TASK: *Ask open-ended questions to learn about what the organization does in terms of serving individuals who are at-risk of opioid overdose. Example questions are below, but adapt them for the type of agency that you are meeting with. For example, you might say "individuals with opioid use disorder" if it's a treatment organization (where people have been diagnosed with OUD) but you might say "people who use opioids" for a social services agency where they may not know if people have been diagnosed with OUD. You can encourage them to think about people who may not realize they are at risk of overdose (e.g., people who use other drugs where fentanyl may be in drug supply), concerned family/friends of people at risk of overdose, and staff who want to be ready to respond to an overdose.*

Can you tell me a little bit about the services that you offer?

Can you tell us about your services for individuals with opioid use disorder?

About how many individuals with OUD does your agency serve in a typical month?

Once you have an understanding of the organization, gauge their interest in learning more about the program and potentially partnering with HCS.

It sounds like you serve people who could benefit from overdose education and naloxone distribution. Would you be interested in hearing more about our Overdose Education and Naloxone Distribution Program?

> ***If yes, present slides 7-12 which explains the OEND program.***
>
> ***If no,*** *close the meeting but leave the door open to future discussions if they change their minds (see last page)*

TASK: *Ask open-ended questions about overdose prevention education at the agency to determine how they could offer OE that meets the SOA requirements.*

Does your agency currently provide overdose prevention education to the [patients/clients/people] that your agency serves?

> *If yes:*
>
> How is overdose education provided? What types of information is typically provided?
>
> Is overdose education provided to all patients/clients? (And if not: Which patients are most likely to receive OE?)

We have a 9 minute overdose education video developed by our team that provides information about how to prevent overdose and how to respond to an overdose using naloxone. The video is also available in Spanish and Arabic, and we have a version with closed captioning for individuals with hearing impairments.

Would a short video be helpful so that more patients/clients could receive overdose education?

> ***If response is "yes,"*** *provide overview of how they can access the HCS Video. Options are:*
>
> - *YouTube if there's wi-fi access*
> - *Download the videos onto their own equipment (e.g., computers, tablets) so that a wi-fi connection isn't required*
> - *HCS can provide an iPad to them where the videos are pre-loaded*
>
> *Then, determine if the agency needs an iPad.* ***The default should be a single tablet for a location unless there is a compelling justification for more than one tablet.*** *If the agency requests multiple tablets, the IF should ensure that multiple individuals will be responsible for training individuals (i.e., that there is a need for multiple tablets based on the program's workflow and intended number of individuals providing OEND). You will need to obtain faculty approval, so do not promise multiple iPads in this meeting. Be sure to document the reasons for needing multiple iPads and how many iPads are necessary.*

If response is "no" because 9 minutes is too long, *discuss if staff could verbally review the HCS Naloxone Brochure which is a trifold brochure that contains information on overdose prevention, overdose recognition, and overdose response. If the staff member verbally reviews the Brochure, it will meet the training requirements of the SOA.*

If response is "no" because they want to use their own overdose education, *discuss whether their training meets the SOA requirements.*

What topics does the overdose education cover? (Mark the topics mentioned below.)
Risk factors of opioid overdose ______
Strategies to prevent opioid overdose ______
Signs of opioid overdose ______
Steps in responding to an overdose ______
Information on naloxone ______
Procedures for administering naloxone ______
Proper storage and expiration of naloxone product dispensed ______

For any topics not checked, ask whether it is included. (e.g., "Does the overdose education cover information about proper storage and expiration of naloxone product being dispensed?")

If there are topics not covered, ask whether the agency would be willing to review a brochure with the patient/client (i.e., the HCS Naloxone Brochure) to ensure that all 7 topics are covered.

Summarize the approach(es) that the agency agrees to use for OE before moving on.
Note: To proceed to implementation, the agency must commit to an OE approach that meets the SOA requirements.

TASK: Discuss naloxone distribution and documentation process.

What would be the best way to ensure that patients receive naloxone quickly after the overdose education is completed?

How many units of naloxone would your agency need in an initial shipment that would meet your agency's needs for about one month?

- *Maximum initial shipment is 72 units (6 cases, 12 units each)*

The last topic we need to discuss are our program's documentation requirements. Our preferred approach is a short electronic survey that is completed in real time for each unit distributed. It's fewer than 10 questions and it is anonymous. The survey just asks about the type of overdose education, for confirmation that the unit of naloxone was distributed, and then basic demographics of the person receiving naloxone. Would that be feasible?

If no, explore why. *Remind them that we can provide an iPad for completing the survey. Remind them also that this is a study and our funding agency, the National Institute on Drug Abuse, needs us to collect this information. It is also how we will know that they are getting low on naloxone so that we can get shipments prepared before they run out.*

Offer paper forms: We can provide a paper version of the form if real-time completion of the survey may not always be feasible (e.g., group OEND, no wi-fi). A single staff person would still need to be responsible for entering the data into the web survey. Would that be feasible?

The agency needs to commit to entering data themselves; we do not have the capacity to enter data for Wave 2 agencies.

WRAP-UP: ***Use the Slides 13-16 in the PowerPoint to set action items and share information about KY-OPEN.***

1) Note that you will be sending the Parter Organizational Planning Tool which briefly summarizes roles of agency staff and HCS staff after today's meeting, and that if they have any revisions, to please just let you know.

2) Be sure to explain that naloxone cannot be sent until we receive the signed Standing Order Agreement.

3) About one week after the naloxone has been sent, you will reach out by email or phone to just check-in about whether they have any questions as they are getting started with implementation. Then about one month after implementation begins, there will be a follow-up meeting.

4) If they are interested, they are also welcome to participate in KY-OPEN (this is not required—just an option). Use slides 15-16 to explain what KY-OPEN is.

5) Express appreciation for everyone's time and convey enthusiasm for the partnership. Close the meeting.

IF ORGANIZATION IS NOT INTERESTED IN PARTNERING WITH US (doesn't offer OE & doesn't want to adopt OE, doesn't need naloxone, is unwilling to meet the data requirements)

Would it be possible to distribute information brochures with overdose education resources to patients and their friends/family being served by your agency?

If yes*, ask about how many HCS Naloxone Brochures they would like and confirm a mailing address for sending them.*

Thank you for making the time to meet with us today. If at some point in the future your team is interested in partnering with us regarding overdose education and naloxone distribution, please let us know.

Standing Order Agreement for OEND

Naloxone HCl is an opioid antagonist indicated for the emergency treatment of known or suspected overdose, as manifested by respiratory or central nervous system depression.

PURPOSE: This standing order agreement is intended to provide the safe, efficient distribution of naloxone within Kentucky communities participating in the HEALing Communities Study (HCS). This agreement is intended for use by HCS staff and community partner agencies to distribute naloxone (including via mail) to any person who is at risk of experiencing an opioid-related overdose or any person who may be in a position to assist a person experiencing an opioid-related overdose. Agencies may also maintain a supply of naloxone for administration within the agency by trained employees and trained volunteer staff.

AUTHORITY: This standing order is issued pursuant to KRS 217.182, which permits a practitioner to dispense or provide a legend drug for a legitimate medical purpose and in the course of professional practice, and KRS 217.186, which specifically protects licensed health-care providers from disciplinary or other adverse action when dispensing or providing naloxone to a person or agency.

DISPENSING PROTOCOL: The dispensing of naloxone to HCS community partner agencies is authorized by this standing order agreement and will be conducted according to the HCS Naloxone Standard Operating Procedure. Partner agencies may distribute naloxone dispensed under this agreement to any person who has completed the required bystander training, as described herein.

REQUIRED TRAINING: Prior to distributing naloxone dispensed under this agreement, partner agencies shall ensure that each person receiving naloxone has completed bystander training in opioid overdose prevention, recognition and response. This training requirement may be met by completion of the online module available or by any training method that includes all of the following elements:

(1) Risk factors of opioid overdose;
(2) Strategies to prevent opioid overdose;
(3) Signs of opioid overdose;
(4) Steps in responding to an overdose;
(5) Information on naloxone;
(6) Procedures for administering naloxone; and
(7) Proper storage and expiration of naloxone product dispensed

MEDICATION: This standing order authorizes the dispensing and distribution of the NARCAN Nasal Spray 4 mg two-pack unit (NDC 69547-0353-02). Each unit of medication will be labeled as follows:

NIH HEAL INITIATIVE
HEALing Communities Study Kentucky

Prescriber:
Dr. Michelle Lofwall
845 Angliana Ave
Lexington, KY 40508
(859) 562-3254

NAME OF AGENCY
For suspected opioid overdose, call 911;
spray contents of 1 device into 1 nostril;
if needed, repeat with additional device(s)
in opposite nostril(s) every 2-3 minutes
NARCAN NASAL SPRAY 4 mg Qty: 2 doses

NIH HEAL INITIATIVE
HEALing Communities Study Kentucky

Prescriber:
Dr. Michelle Lofwall
845 Angliana Ave
Lexington, KY 40508
(859) 562-3254

FOR ON-SITE USE ONLY

NAME OF AGENCY
For suspected opioid overdose, call 911;
spray contents of 1 device into 1 nostril;
if needed, repeat with additional device(s)
in opposite nostril(s) every 2-3 minutes
NARCAN NASAL SPRAY 4 mg Qty: 2 doses

DOCUMENTATION: All units of naloxone dispensed and distributed under this agreement will be documented according to the HCS Naloxone Standard Operating Procedure outlined in the Naloxone Procedures for Partner Agencies Manual. Additionally, the individual distributing naloxone at the HCS partner agency shall complete and submit the electronic survey with de-identified demographic information about naloxone recipients upon each distribution of naloxone. The HCS partner agency agrees to keep a list of all persons trained on overdose education and naloxone distribution who are authorized to administer naloxone under this agreement.

EFFECTIVE DATE: This agreement shall take effect immediately upon signing and shall remain in effect for the duration of HCS. At the conclusion of HCS, units of naloxone that have been dispensed under this agreement may be distributed by the partner agency until supplies are exhausted or the expiration date of the naloxone units, whichever comes first.

AUTHORIZATION:
I, **Michelle R. Lofwall, M.D.**, a licensed physician in the state of Kentucky, authorize the dispensing of naloxone to HCS community partner agencies as described herein. I further authorize partner agencies to distribute naloxone according to this agreement as well as to maintain supplies of naloxone to fulfill the needs of the community and HCS.

____ _________________

Michelle R. Lofwall, M.D. Date

I, _____________________, a representative of HCS partner agency _____________________, agree that all naloxone received from HCS will be distributed in accordance with this agreement and in compliance with KRS 217.186. I further authorize any individual employed by or volunteering in the HCS partner agency who has been trained in overdose response to administer naloxone received from HCS to an individual experiencing an overdose or suspected overdose on-site, and explicitly authorize the following individuals to serve as representatives eligible to receive and distribute HCS naloxone on behalf of the partner agency:

__________________________________ __________________________________

__________________________________ __________________________________

__________________________________ __________________________________

__________________________________ __________________________________

Revisions to this list of authorized representatives may be made by email to naloxoneHCS@uky.edu or by fax to 859-562-3356.

____

Signature

____ _________________

Name and title Date

HCS Naloxone Video for Overdose Education
YouTube Links

English: https://www.youtube.com/watch?v=hF-iZUkkdt0

English with closed captioning: https://www.youtube.com/watch?v=Ap6Eyco5gjQ

Spanish: https://www.youtube.com/watch?v=qiFDuknpT1U

Arabic: https://www.youtube.com/watch?v=AG8r9h300TU

Swahili: https://www.youtube.com/watch?v=tvub0nTqnf8

You may be at increased risk of opioid overdose:

- If you are sick or have acute or chronic medical problems
- If you mix drugs or use alone
- If you have recently experienced opioid withdrawal
- If you drink alcohol heavily or smoke

To reduce the risk of overdose:

- Always use with a friend or around other people, never alone
- Try to buy from the same dealer. If you can't, talk to others who have bought from that dealer, and test the strength of the drug before you do the whole amount
- Avoid mixing drugs, especially opioids or other drugs with alcohol
- Take prescriptions only as directed
- Develop an overdose plan with friends or partners

Now that you have naloxone

Tell someone where it is and how to use it!

For locations to purchase naloxone visit:

http://HealTogetherKy.org

Scan the QR code to watch the How To Use Naloxone educational video:

Common opioids

PRESCRIPTION DRUGS	
**Generic**	**Brand Name**
**Buprenorphine**	Suboxone, Subutex, Zubsolv, Butrans
**Codeine**	Tylenol with Codeine, Tylenol #3
**Fentanyl**	Duragesic
**Hydrocodone**	Vicodin, Lortab, Norco
**Hydromorphone**	Dilaudid
**Oxycodone**	Percocet, OxyContin, Roxicodone, Xtampza ER
**Meperidine**	Demerol
**Methadone**	Dolophine, Methadose
**Morphine**	MSContin
**Oxymorphone**	Opana

ILLICIT DRUGS
**Fentanyl, carfentanil, and related chemicals**
**Heroin**

Other illicit drugs may be tainted with opioids, even if they look like prescription drugs or you are told they are something else.

NIH HEAL INITIATIVE

HEALing Communities Study Kentucky

Opioid Overdose Recognition and Response

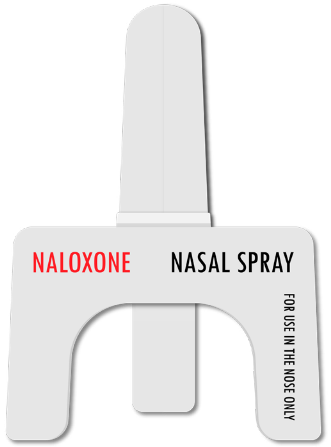

A GUIDE FOR CONCERNED COMMUNITY BYSTANDERS

HEALing Communities Study — Kentucky

Is it an overdose?

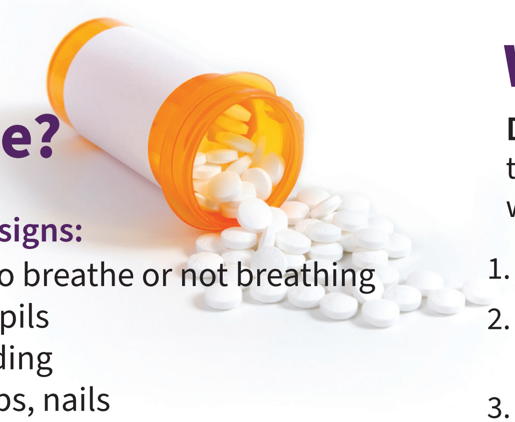

Look for these signs:

- Struggling to breathe or not breathing
- Pinpoint pupils
- Not responding
- Blue/gray lips, nails
- Skin pale, clammy
- Choking, snoring, gurgling sounds

How to give naloxone:

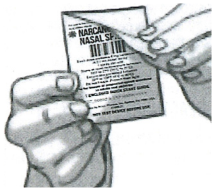

Peel

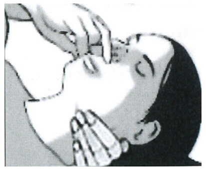

Place

Caution: do not activate (press) device until inserted into the nostril and you are ready to administer as all of the medication will be lost

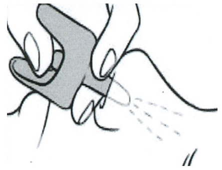

Press

Naloxone Storage & Handling:

- Store naloxone at room temperature between 59°F to 77°F (or for *short* periods of time between 39°F to 104°F)
- Do not freeze naloxone
- Keep naloxone in its box until ready to use and protect from light
- Replace naloxone before the expiration date on the box

What do I do?

Don't panic! Try to wake the person by yelling their name and rubbing the middle of their chest with knuckles (sternal rub)

1. **Call 911**!
2. **Lay person on their back / begin rescue breathing** if breathing is stopped or very slow
3. **Peel back the tab** with the circle to open and remove naloxone from box.
4. Hold naloxone with your thumb on bottom of plunger and your first and middle fingers on either side of nozzle
5. Tilt person's head back; provide support under neck with your hand. **Gently insert tip of nozzle into one nostril** until your fingers on either side of nozzle are against the bottom of person's nose
6. **Press plunger firmly** to give dose of naloxone
7. Remove sprayer from nostril after giving dose
8. Continue rescue breathing if slow / no breathing
9. **IF AFTER 2-3** minutes person is still unresponsive with slow/no breathing repeat steps 3-8 above
10. **Roll person on side*** if breathing on own; continue to monitor breathing; begin rescue breathing again if required. **STAY with person until EMS arrives!**

***Putting someone in the recovery position will keep their airway clear and open, and ensures that any vomit or fluid won't cause them to choke.**

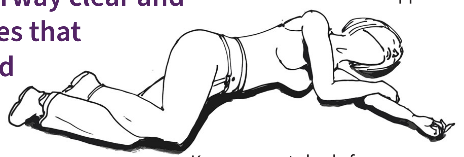

How to perform rescue breathing

Follow these steps for rescue breathing:

1. Place person on their back.
2. Tilt their chin up to open the airway.
3. Check to see if there is any object in their mouth blocking airway, such as gum, toothpick, undissolved pills, syringe cap, cheeked fentanyl patch (these things have ALL been found in the mouths of overdosing people!). If so, remove object.
4. Use a breathing shield/barrier if you have one. Plug their nose with one hand and give two even, regular-sized breaths. Blow enough air into their lungs to make their chest rise. (*Note*: the stomach/abdomen should NOT rise.) If you don't see their chest rise out of the corner of your eye, tilt the head back more and make sure you're plugging their nose.
5. Breathe again. Give one breath every 5 seconds.

Precautions you can take

If possible, follow these safety measures when administering naloxone:

1. Call 911
2. Wear gloves and a face covering
3. Wash hands or use hand sanitizer before and after administration
4. Cough or sneeze into arm

Puede tener un mayor riesgo de sobredosis de opioides:

- Si está enfermo o tiene problemas médicos agudos o crónicos.
- Si mezcla fármacos o los consume cuando está solo.
- Si ha experimentado recientemente abstinencia de opioides.
- Si toma mucho alcohol o fuma.

Para reducir el riesgo de sobredosis:

- Consúmalos siempre con un amigo o cerca de otras personas, nunca solo.
- Trate de comprar al mismo distribuidor. Si no puede, hable con otras personas que le hayan comprado a ese distribuidor y pruebe la potencia del fármaco antes de consumir toda la cantidad.
- Evite mezclar fármacos, especialmente opioides u otras drogas con alcohol.
- Tome los medicamentos recetados solo según las indicaciones.
- Desarrolle un plan en caso de sobredosis con ayuda de amigos o compañeros

Ahora que tiene naloxona

¡Dígale a alguien dónde se encuentra y cómo usarla!

Para conocer los lugares donde comprar naloxona, visite:

http://HealTogetherKy.org

Escanee el código QR para ver el video educativo Cómo usar la naloxona:

Opioides comunes

MEDICAMENTOS RECETADOS	
**Genéricos**	**Marca**
**Buprenorfina**	Suboxone, Subutex, Zubsolv, Butrans
**Codeína**	Tylenol con codeína, Tylenol No. 3
**Fentanilo**	Duragesic
**Hidrocodona**	Vicodin, Lortab, Norco
**Hidromorfona**	Dilaudid
**Oxicodona**	Percocet, OxyContin, Roxicodone, Xtampza ER
**Meperidina**	Demerol
**Metadona**	Dolophine, Methadose
**Morfina**	MSContin
**Oximorfona**	Opana

DROGAS ILÍCITAS
**Fentanilo, carfentanilo y productos químicos relacionados**
**Heroína**

Otras drogas ilícitas pueden estar contaminadas con opioides, incluso si parecen medicamentos recetados o si le dicen que son otra cosa.

NIH HEAL INITIATIVE

HEALing Communities Study Kentucky

Reconocimiento y respuesta ante casos de sobredosis de opioides

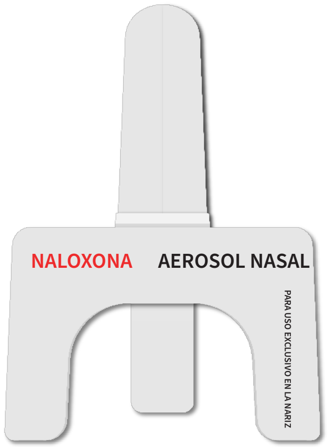

UNA GUÍA PARA ESPECTADORES PREOCUPADOS DE LA COMUNIDAD

HEALing Communities Study — Kentucky

¿Es una sobredosis?

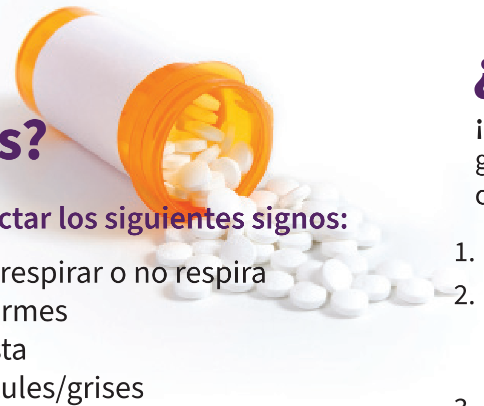

Observe para detectar los siguientes signos:

- Dificultad para respirar o no respira
- Pupilas puntiformes
- No hay respuesta
- Labios, uñas azules/grises
- Piel pálida, sudada
- Sonidos de asfixia, ronquidos o gorgoteo

Cómo administrar naloxona:

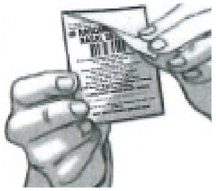

Retirar

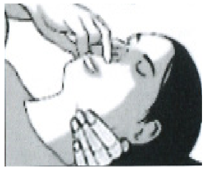

Colocar

Precaución: no active (presione) el dispositivo hasta que se inserte en la fosa nasal y esté listo para administrar, ya que se perderá todo el medicamento.

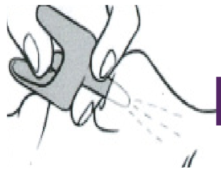

Presionar

Almacenamiento y manipulación de la naloxona:

- Almacene la naloxona a temperatura ambiente entre 59 °F a 77 °F (o por períodos *cortos* entre 39° F a 104 °F)
- No congele la naloxona
- Mantenga la naloxona en su caja hasta el momento de usar y protéjala de la luz.
- Reemplace la naloxona antes de la fecha de vencimiento que aparece en la caja.

¿Qué debo hacer?

¡No entre en pánico! Intente despertar a la persona gritando su nombre y frotándole el centro del pecho con los nudillos (fricción del esternón).

1. **¡Llame al 911**!
2. **Acueste a la persona boca arriba/comience la respiración asistida** si esta dejó de respirar o su respiración es muy lenta.
3. **Retire la pestaña** con el círculo para abrir y retire la naloxona de la caja.
4. Sostenga la naloxona con el pulgar en la parte inferior del émbolo y los dedos índice y medio a cada lado de la boquilla.
5. Incline la cabeza de la persona hacia atrás; apoye su cuello sobre en su mano. **Inserte cuidadosamente la punta de la boquilla en una fosa nasal** hasta que sus dedos a cada lado de la boquilla estén contra la parte inferior de la nariz de la persona.
6. **Presione el émbolo firmemente** para administrar la dosis de naloxona.
7. Retire el aerosol de la fosa nasal después de administrar la dosis.
8. Continúe con la respiración asistida si respira lentamente o no respira.
9. **SI DESPUÉS DE 2 A 3** minutos la persona aún no responde y su respiración es lenta o no respira, repita los pasos 3 a 8 anteriores.
10. **Gire a la persona sobre su lado*** si respira por sí mismo; continúe monitoreando la respiración; comience de nuevo con la respiración asistida si es necesario. **¡QUÉDESE con la persona hasta que llegue el servicio de emergencias médicas (EMS)!**

---

*Poner a alguien en la posición de recuperación mantendrá sus vías respiratorias despejadas y abiertas y garantizará que el vómito o los líquidos no hagan que se ahogue.

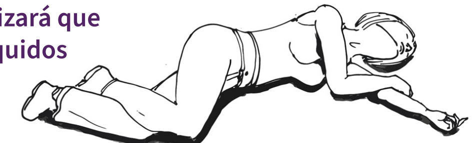

Cómo realizar la respiración asistida

Siga estos pasos para la respiración asistida:

1. Coloque a la persona boca arriba.
2. Incline su barbilla hacia arriba para abrir las vías respiratorias.
3. Verifique si hay algún objeto en la boca que bloquee las vías respiratorias, como goma de mascar, palillo de dientes, píldoras sin disolver, tapa de jeringa, parche de fentanilo en la mejilla (¡TODAS estas cosas se han encontrado en la boca de personas con sobredosis!). Si es así, retire el objeto.
4. Utilice un protector/barrera de protección para reanimación cardiopulmonar si tiene uno. Tape la nariz de la persona con una mano y administre dos respiraciones uniformes y regulares. Sople suficiente aire en sus pulmones para que su pecho se eleve. (*Nota*: el estómago/abdomen NO debe elevarse). Si no ve que su pecho se eleve por el rabillo del ojo, incline más la cabeza hacia atrás y asegúrese de taparle la nariz.
5. Vuelva a administrar las respiraciones. Administre una respiración cada 5 segundos.

Precauciones que puede tomar

Si es posible, siga estas medidas de seguridad cuando administre la naloxona:

1. Llame al 911
2. Use guantes y una mascarilla para cubrir su rostro.
3. Lávese las manos o use desinfectante para manos antes y después de la administración.
4. Tosa o estornude en el brazo.

قد تكون أكثر عرضةً لخطر التعرض لجرعة زائدة من المواد الأفيونية في الحالات الآتية:

- إذا كنت مريضًا أو تعاني مشكلات طبية حادة أو مزمنة
- إذا قمت بخلط العقاقير أو المخدرات أو تعاطيها منفردًا
- إذا كنت قد عانيت مؤخرًا أعراضًا انسحابية للمواد الأفيونية
- إذا كنت تشرب الكحوليات بشراهة أو تدخن

لتقليل خطر التعرض للجرعة الزائدة:

- احرص دائمًا أن تكون مع أحد الأصدقاء أو بجوار أحد الأشخاص ، إذا كنت بصدد تعاطي أو تناول المُخدرات وتجنب التواجد بمُفردك.
- حاوِل الشراء من التاجر نفسه. إذا لم تستطع ذلك، تحدث إلى الآخرين الذين اشتروا من ذلك التاجر، واختبر مدى قوة مفعول العقار قبل أن تتناول الجرعة بأكملها
- تجنب خلط العقاقير، لاسيما المواد الأفيونية أو غيرها من العقاقير مع الكحول
- تناوَل الوصفات الطبية فقط حسب التوجيهات
- ضع خطة مع الأصدقاء أو الزملاء للتصرف في حالة التعرض لجرعة زائدة

الآن بعد أن أصبح لديك دواء نالوكسون (Naloxone)

أخبِر شخصًا ما بمكانه وعن كيفية استخدامه

لمعرفة منافذ شراء دواء نالوكسون (Naloxone)، تفضل بزيارة موقع الويب:

http://HealTogetherKy.org

يمكنك مسح رمز الاستجابة السريعة (QR) ضوئيًا لمشاهدة مقطع الفيديو التعليمي "كيفية استخدام دواء نالوكسون (Naloxone)":

المواد الأفيونية الشائعة

العقاقير الموصوفة طبيًا

عامة	اسم العلامة التجارية
**البوبرينورفين (Buprenorphine)**	السوبوتيكس (Suboxone)، السوبوتكس (Subutex)، الزوبسلو (Zubsolv)، البوترانس (Butrans)
**الكودين (Codeine)**	التايلينول (Tylenol)، مع الكودين (Codeine)، التايلينول رقم 3 (Tylenol #3)
**الفنتانيل (Fentanyl)**	ديورجيسيك (Duragesic)
**الهيدروكودون (Hydrocodone)**	الفيكودين (Vicodin)، اللورتاب (Lortab)، النوركو (Norco)
**الهيدرومورفين (Hydromorphone)**	الديلاوديد (Dilaudid)
**الأوكسيكودون (Oxycodone)**	البيركوسيت(Percocet)، الأوكسيكودون(Oxycodone)، الروكسيكودون(Roxicodone)، الإكستامبزا إيه آر (Xtampza ER)
**الميبيريدين (Meperidine)**	الديميرول (Demerol)
**الميثادون (Methadone)**	الدولوفين (Dolophine)، الميثادون (Methadone)
**المورفين (Morphine)**	إم إس كونتين (MSContin)
**الأوكسي مورفون (Oxymorphone)**	الأوبانا (Opana)

العقاقير غير المشروعة

الفنتانيل (Fentanyl) والكارفينتانيل (carfentanil) والمواد الكيميائية ذات الصلة

الهيروين (Heroin)

قد تكون العقاقير غير المشروعة الأخرى مغشوشة بالمواد الأفيونية، حتى لو كانت تبدو مثل الأدوية الموصوفة طبيًا أو قيل لك إنها شيء آخر.

NIH
HEAL
INITIATIVE

HEALing Communities Study
Kentucky

التعرّف على حالة الجرعة الزائدة من المواد الأفيونية والتصرف إزاءها

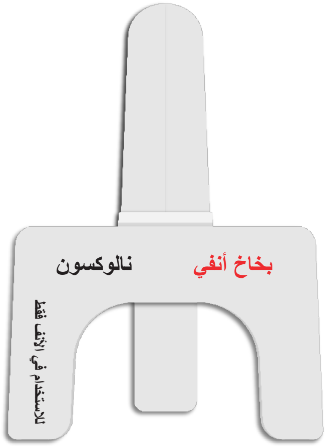

دليل لمن يهمه الأمر

من الأفراد العاديين في المجتمع

HEALing Communities Study — Kentucky

ما الجرعة الزائدة؟

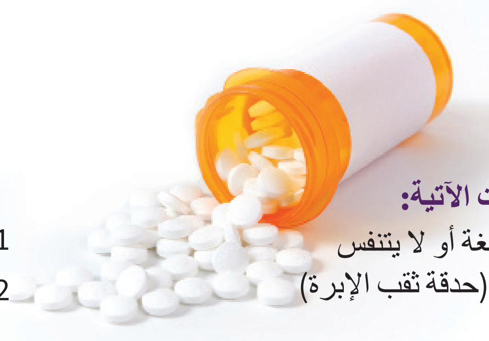

ابحث عن هذه العلامات الآتية:

- يتنفس بصعوبة بالغة أو لا يتنفس
- تضيُّق حدقة عينه (حدقة ثقب الإبرة)
- عدم الاستجابة
- تغيُّر لون الشفاه والأظافر إلى اللون الأزرق/الرمادي
- شحوب الجلد والتعرق
- إصدار أصوات اختناق أو شخير أو غرغرة

كيفية إعطاء دواء نالوكسون (Naloxone):

إزالة الغلاف

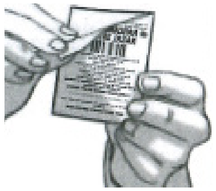

وضع الفوهة في الأنف

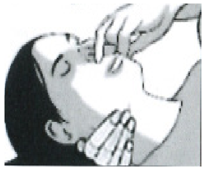

تحذير: لا تضغط على مكبس البخاخ قبل أن تدخل الفوهة في فتحة أنف المريض وتكون جاهزًا لإعطائه، وإلا ستُهدر جرعة الدواء بأكملها

الضغط على المكبس

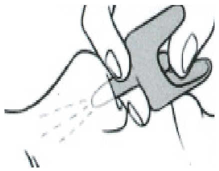

تخزين النالوكسون (Naloxone) وحفظه:

- احرص على تخزين دواء نالوكسون (Naloxone) في غرفة بدرجة حرارة من 59° درجة فهرنهايت إلى 77° درجة فهرنهايت (أو لفترات زمنية قصيرة من 39° درجة فهرنهايت إلى 149° درجة فهرنهايت)
- لا تجمِّد دواء نالوكسون (Naloxone)
- احتفظ بدواء نالوكسون (Naloxone) في عبوته حتى تكون جاهزًا لاستخدامه، واحمه من الضوء
- استبدل دواء نالوكسون (Naloxone) قبل تاريخ انتهاء الصلاحية المكتوب على العبوة

كيف أتصرف حيالها؟

لا داعي للذعر! حاول إفاقة المريض بالصراخ تجاهه مناديًا باسمه وفرك منتصف صدره بمفاصل أصابعك (فرك منطقة الصدر)

1. **الاتصال على رقم 911!**
2. **ضع الشخص مستلقيًا على ظهره/ابدأ في إجراء التنفس الإنقاذي** إذا توقفت أنفاسه أو كانت بطيئة للغاية
3. **قم بإزالة الغلاف الخلفي** من عند علامة الدائرة لفتح عبوة دواء نالوكسون (Naloxone) وإخراجه منها.
4. أمسِك دواء نالوكسون (Naloxone) مع وضع إبهامك على أسفل المكبس وإصبعيك السبابة والوسطى على جانبَي الفوهة
5. قم بإمالة رأس الشخص المريض إلى الخلف؛ ضع يدك أسفل رقبته وادفعها لأعلى. **أدخِل طرف الفوهة برفق في إحدى فتحتَي أنفه** حتى يصبح إصبعيك على جانبَي الفوهة ملامسَين للجزء السفلي من أنف الشخص
6. **اضغط على المكبس بقوة** لإعطاء المريض جرعة كاملة من دواء نالوكسون (Naloxone)
7. أخرج البخاخ من فتحة أنفه بعد إعطاء الجرعة
8. استأنف إجراء التنفس الإنقاذي إذا كان تنفسه بطيئًا/لا يتنفس
9. إذا لم يستجب المريض **بعد 3-2 دقائق** وكان يعاني من بطء في التنفس/ أو لا يتنفس مُطلقًا، كرر الخطوات من 3 إلى 8 الواردة أعلاه
10. **قم بلف الشخص وتثبيته على جانبه*** إذا كان يتنفس من تلقاء نفسه؛ استمر في مراقبة أنفاسه. ابدأ في إجراء التنفس الإنقاذي مرة أخرى إذا لزم الأمر. **ابقَ مع الشخص المصاب لحين وصول موظفي خدمات الطوارئ الطبية!**

***إن وضع الشخص المريض مستلقيًا بوضعية الإفاقة سيحافظ على مجرى الهواء لديه خاليًا ومفتوحًا ويضمن ألا يؤدي أي قيء أو سائل إلى اختناقه.**

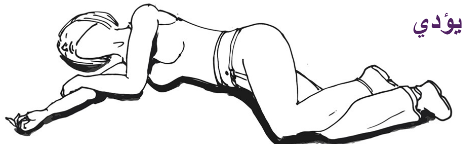

كيفية إجراء التنفس الإنقاذي (إعطاء أنفاس للمريض بالنفخ في فمه)

اتبع الخطوات الآتية لإجراء التنفس الإنقاذي للمريض:

1. ضع الشخص مستلقيًا على ظهره.
2. قم بإمالة ذقنه لأعلى لفتح مجرى الهواء لديه.
3. تحقق لمعرفة ما إذا كان هناك أي شيء في فمه يسد مجرى الهواء لديه، كعلكة أو عود أسنان أو حبوب غير ذائبة أو غطاء محقنة أو رقعة الفنتانيل اللاصقة (عُثر على مثل هذه الأشياء جميعًا في أفواه ضحايا الجرعات الزائدة!). إذا كان الأمر كذلك، أخرج هذا الشيء!
4. استخدم واقي/حاجز التنفس إذا كان لديك واحد. قم بسد أنفه بإحدى يديك وامنحه نفسين متساويين بكميات عادية. انفخ كمية كافية من الهواء في رئتيه ليرتفع صدره. (*ملحوظة*: لا ينبغي أن ترتفع المعدة/البطن). إذا كنت لا ترى صدره يرتفع بطرف عينك، فقم بإمالة رأسه إلى الخلف أكثر وتأكد من أنك تسد أنفه.
5. أعطه أنفاسًا مرة أخرى. أعطه نفسًا واحدًا في كل مرة لمدة 5 ثوان.

الاحتياطات التي يمكنك اتخاذها

إن أمكن، اتبع تدابير السلامة الآتية عند إعطاء دواء نالوكسون (Naloxone):

1. الاتصال على رقم 911
2. ارتداء قفازات وغطاء للوجه
3. غسل اليدين أو استخدام معقم لليدين قبل إعطاء الدواء وبعده
4. تغطية الفم بالذراع عند العطس أو السعال

NIH
HEAL
INITIATIVE
HEALing Communities Study
Kentucky

Frequently Asked Questions (FAQ) about the
HCS Overdose Education and Naloxone Distribution (OEND) Program

1. STANDING ORDER AGREEMENT

What is a standing order agreement (SOA)?
A standing order agreement (SOA) is a written care directive from an authorized medical provider that delineates the circumstances under which another person may act to carry out specific medical orders. The SOA waives the usual requirement of the need for a treatment relationship between the patient and the authorized medical provider prior to the execution of a medical order. The ability to use SOAs for dispensing naloxone is specifically addressed in Kentucky state law (KRS 217.186).

For HCS, the standing order agreement is between our study physician, Dr. Michelle Lofwall, and your organization. You will be asked to identify in the SOA the staff members who will distribute HCS-provided naloxone. The SOA then gives these staff members the authority to distribute naloxone to individuals if those individuals have completed overdose education that meets certain training requirements.

What are the liability issues related to naloxone distribution?
The HCS standing order agreement (SOA) and our procedures for dispensing naloxone have been reviewed and approved by staff from the Kentucky Board of Medical Licensure. Kentucky state law (KRS 217.186) provides the legal basis for naloxone distribution, and the law includes the following:

> "A person or **agency**, including a peace officer, jailer, firefighter, paramedic, or emergency medical technician or a school employee authorized to administer medication under KRS 156.502, may:
> (a) Receive a prescription for an opioid antagonist [naloxone];
> (b) Possess an opioid antagonist [naloxone] pursuant to this subsection and any equipment needed for its administration; and
> (c) Administer an opioid antagonist [naloxone] to an individual suffering from an apparent opioid-related overdose; and
> (d) Provide, as part of a harm reduction program, an opioid antagonist to persons who have been trained on the mechanism and circumstances of its administration."

In Section 5 of KRS 217.186, the law further states:

> "A person acting in good faith who provides or administers an opioid antagonist [naloxone] received under this section shall be immune from criminal and civil liability for the provision or administration, unless personal injury results from the gross negligence or willful or wanton misconduct of the person providing or administering the drug."

If you are interested in reading Kentucky naloxone's law, we will send it to you.

Furthermore, liability issues are low because naloxone has no potential for abuse, it has a low risk of adverse reactions, and it has no significant clinical effect if opioids are not present. These three facts have led key experts to conclude the following: "*Because harm to the patient is a necessary component of a successful lawsuit, this translates into a low risk of civil liability for naloxone prescribing or*

dispensing. Indeed, legal experts believe that prescribing naloxone does not increase liability risk, and ***a recent review did not find a single lawsuit anywhere in the United States related to the outpatient prescription or dispensing of naloxone.*** *"* (from Davis, C. & Carr, D. (2017). State legal innovations to encourage naloxone dispensing. Journal of the American Pharmacists Association, *57,* S180-S184.) Please let us know if you would like us to provide this article to you.

If we have a medical provider on staff, should we enter into a SOA with HCS?
If your organization has a medical provider on staff, we can set up an agreement using the process of Internal Prescriptive Authority (IPA). Through this IPA model, we can help your organization to set up an SOA under your provider's medical license, and then your organization can receive no-cost naloxone that is shipped directly from Emergent Biosolutions. Under this IPA model, your organization will need to assume responsibility for labeling the units of naloxone prior to distributing them to individuals.

What is the advantage of a SOA/IPA with HCS instead of relying on our prescriber to prescribe naloxone to the individuals who we serve?
The HCS SOA/IPA facilitates the provision of no-cost naloxone to your organization, which may reduce costs (e.g., co-pays) that a person would incur if he or she were filling a naloxone prescription at a pharmacy. There is a risk that naloxone prescriptions are not filled. We also know that some communities have pharmacies where naloxone is not available. The HCS SOA/IPA model overcomes some of these barriers to naloxone access.

Can our SOA include another organization?
If your organization is partnering with another organization, we require that separate SOAs be set up with each organization.

How does your standing order agreement (SOA) relate to the documents we signed for KORE (Kentucky Opioid Response Effort; the state investment of federal money from SAMHSA to address the opioid crisis in Kentucky)?
The HCS SOA represents an agreement between our study physician, Dr. Michelle Lofwall, and your organization, so it does not relate to any documents that you have already signed for KORE.

2. STORING AND RECEIVING HCS NALOXONE

How must the naloxone provided by HCS be stored?
Our standing order agreement (SOA) requires that the naloxone is securely stored. Because naloxone is not a controlled substance, a locked desk drawer or locked cabinet is sufficient. If your organization already stores medication samples, the security requirement for naloxone is similar. Naloxone does not need to be refrigerated but should not be exposed to extreme heat or cold.

How quickly will we receive our HCS naloxone?
Once the standing order agreement has been signed by your organization and by the HCS physician, we can usually ship naloxone to your organization so that it arrives within 2 weeks.

How do we receive the shipments? Do we have to pick them up?
You do not have to pick them up. Depending on the distance between Lexington and your organization, HCS will either ship the naloxone to you via FedEx or an HCS staff member will deliver the naloxone to your organization.

How often will we receive shipments?
Our goal is to ship enough naloxone to your organization on a quarterly basis to meet your needs. Our HCS Naloxone Coordinator monitors the number of units of naloxone that your organization has distributed, so we can ship more naloxone if needed before the next quarter.

What should we do if we run out of naloxone?

If you are running out of naloxone, please contact your Implementation Facilitator or email our HCS Naloxone Coordinator (naloxoneHCS@uky.edu), who will work as quickly as possible to re-supply your organization, either by shipping via FedEx or by traveling to your organization to deliver it. To avoid a disruption in supply, please let your Implementation Facilitator know if you are running low; do not wait until the supply has been depleted.

What happens if we don't use all of our naloxone? Will we still receive a shipment?
Our HCS Naloxone Coordinator is monitoring the naloxone that your organization has distributed. If your organization has a large remaining supply of naloxone around the time of the quarterly shipment, we will reach out and reschedule when we will ship more naloxone to your organization.

3. DISTRIBUTING NALOXONE

Who from our organization can distribute naloxone?
Any staff person who is listed on the standing order agreement can distribute naloxone, so long as they are also ensuring the naloxone recipient has completed the overdose education. The staff distributing naloxone do not need to be medical providers. We recommend identifying multiple staff within your organization who can distribute naloxone so that as many people can be reached as possible. If there are changes in the staff who are involved in OEND (e.g., new staff member, staff member leaves the agency), please notify your Implementation Facilitator immediately.

Who can receive the naloxone?
Any patient or client served by your organization who completes overdose education can receive naloxone. You may also distribute naloxone to family members or community members if they complete the overdose education. Please see the section on "Overdose Education" for more details. Staff can also receive naloxone to carry and administer as a Good Samaritan if they complete the overdose education.

If our staff do the overdose education training, can they receive naloxone?
Yes. If your staff member completes the overdose education training, he or she can receive a unit of naloxone (which contains two doses) to carry and administer as a Good Samaritan during her or his daily activities.

Can we give naloxone to minors?
Naloxone can be given to any individual served by your organization who has completed the training and who is deemed as capable of administering naloxone during an overdose. Kentucky's naloxone law does not include a minimum age to receive naloxone.

What happens if someone is enrolled in our program but lives in another county?
As long as your program is in an HCS county, you are able to provide naloxone to someone who is being served by your program who lives in another county.

If a person who has received overdose education and a unit of naloxone returns to our agency and asks for another unit of naloxone, what should we do?
If the person has previously completed the overdose education and comes back for another unit of naloxone, please distribute another unit of naloxone and then record the distribution in the electronic survey. So long as the person has previously completed the overdose education, your agency is not required to re-train the person.

Can we give naloxone from HCS to other organizations with which we work, so that they can distribute to their clients?
No. Our standing order agreement only allows for your organization to train an individual and give the naloxone to that individual. If there is another organization that would benefit from HCS-provided naloxone, please let us know and we will work to set up a standing order agreement with them.

We are concerned about the possibility that overdoses may occur on the premises of our agency. What should we do?
If your agency is concerned about the possibility that overdoses may occur on your premises, we recommend training all staff in overdose education and maintaining a separate supply of naloxone for on-site administration. HCS can provide your agency with no-cost units of naloxone that are specifically labeled for on-site administration and stored in a central location if all staff are trained in overdose education (e.g., watch the HCS Naloxone Video). Any trained staff member can then use naloxone labeled for on-site administration if an overdose occurs at your agency. Trained agency staff who may administer naloxone during an on-site overdose but who are not involved in distributing naloxone to people served by your agency do not need to be listed on the Standing Order Agreement (SOA). If your agency hires new staff, your agency will need to train the new staff in overdose education so that they can respond to an on-site overdose.

4. OVERDOSE EDUCATION

What are our options for overdose education?
HCS is offering an overdose education video that is 9 minutes and covers all of the overdose education requirements in the standing order agreement. HCS can provide one or more iPads to help with implementing the overdose education video, although it can also be shown using your existing equipment (i.e., computer, iPad). This video is also posted on YouTube at:
https://www.youtube.com/watch?v=IZMIeZybx_Q.

Your organization can also choose to provide one-on-one or group overdose education, as long as that training covers the seven components of the HCS standing order agreement. Those seven topics are:

(1) Risk factors for opioid overdose
(2) Strategies to prevent opioid overdose
(3) Signs of opioid overdose
(4) Steps in responding to an overdose
(5) Information on naloxone
(6) Procedures for administering naloxone
(7) Proper storage and expiration of naloxone product dispensed

If your organization's current overdose education covers some but not all of those seven topics, we can provide a brochure that covers all seven topics. If the brochure is reviewed with the individual receiving the naloxone, that would meet the requirements of our standing order agreement.

Can we deliver overdose education (e.g., videos, brochure review, other training that meets the seven components) in a group and then fill out the demographic form individually?
Yes. The HCS standing order agreement only requires that individuals are trained, not the format of the training.

We have not had an education component in the past. Won't this slow down our efforts to put naloxone in people's hands?
Research has shown that individuals who have been trained to use naloxone are more likely to correctly use naloxone when responding to a person who is overdosing. HCS is offering supports for education--a 9-minute video or a brochure that, if reviewed with the recipient, will meet the education requirement of the HCS standing order agreement.

Can we just provide individuals with a brochure to meet the education requirement?
Providing a brochure, without reviewing the brochure with the individual, does <u>not</u> meet the education requirement of the standing order agreement. Overdose education is important because research has shown that trained individuals are more likely to correctly use naloxone when responding to a person who is overdosing than those who have not been trained.

How do I access the HCS Naloxone Video for overdose education?

There are two ways to access the HCS Naloxone Video. First, the HCS Naloxone Video is already loaded onto the home screen of your iPad and can be accessed without being connected to the internet. The HCS Naloxone Video is also posted on YouTube at: https://www.youtube.com/watch?v=IZMleZybx_Q

What if an individual needs OEND but does not speak English or has a hearing impairment?
The HCS Naloxone Video has been translated into Spanish and Arabic. Also, the HCS Naloxone Video is available with closed captioning to train individuals with hearing impairments. On most HCS iPads, these videos should be already loaded and can be found on the home screen of the iPad in a folder titled "Other Translations". If your iPad is missing any of these translated videos or the version with closed captioning, please let your Implementation Facilitator know, and we will arrange to get the video added to your iPad. If you need an additional language, please let your Implementation Facilitator know and we will do our best to translate the HCS Naloxone Video into that language.

5. iPAD

Our OEND iPad was stolen. What should we do?
Please notify your Implementation Facilitator as soon as possible, and they will send you the make, model, and serial number of the iPad, which will be needed by your local police department. Please then report the theft to your local police department and ask for a copy of the police report. Once you have received a copy of the police report, please send that report to your Implementation Facilitator. Your Implementation Facilitator will then work with you to get a replacement iPad as soon as possible.

I need help using the electronic survey to enter data. Is there a video recording of the training provided by the HCS Naloxone Program Manager?
Yes, our Naloxone Program Manager, Josie Watson, has developed a very short video training that can be used as a refresher about entering data using the electronic survey. If you would like to view the video recording about how to use the electronic survey, please visit: https://www.youtube.com/watch?v=bCJzjYUuHz8. We realize that you still may have questions, so please also feel free to reach out to your Implementation Facilitator with questions.

I'm having trouble entering our data using the electronic survey. What should I do?
If you are having trouble with the survey, please contact your Implementation Facilitator or the HCS Naloxone Program Manager, Josie Watson, via email at naloxoneHCS@uky.edu.

Do I always need to be connected to the internet to use the iPad for OEND?
If you do not have access to the internet, you can still use the iPad to show the HCS Naloxone Video.

- To show the HCS video when you are not connected to the internet, simply click on "HCS Naloxone Video" on the home screen of your iPad.

You do need access to the internet in order to enter distribution and demographic data. However, if you anticipate that there will be times when internet is not available, we can provide paper forms that then can be entered by a staff member once they are back in an environment with internet.

I do not have access to WiFi at my agency. What should I do?
HCS can provide an ethernet adapter that will allow the iPad to be plugged into an ethernet port which will connect your iPad to the internet. Please email your Implementation Facilitator, who will order the adapter and will work with you to ensure that the iPad can be connected to the internet using the adapter.

I can't get my iPad to turn on. What should I do?
Two things to consider when the iPad is not working:

1) Does the iPad need to be charged? If you plug it into the charger, does a lightning bolt symbol appear? If there's a lightning bolt symbol, that indicates that the iPad is now receiving power. Please give the iPad some time to recharge.

2) Have you made sure to push the power button? The power button is located on the right side of the iPad.

If your iPad still will not turn on, please contact your Implementation Facilitator.

My iPad says that it needs updates. Should I install the updates?
Yes, please install any updates if the iPad asks you to do so. Please note that your iPad may need to be charged to <u>at least 20% power</u> in order to install updates successfully.

Can we receive more than one iPad for the overdose education videos?
Yes, if your organization plans to have multiple staff delivering overdose education to the individuals served by your organization, HCS can provide more than one iPad if it is necessary. If multiple iPads will help your organization deliver overdose education and distribute naloxone to more individuals, we are happy to provide them.

Can we receive an iPad with the educational videos if we do not need HCS to provide the naloxone?
Yes. The HCS team is happy to provide an iPad so that your organization can use one of the educational videos with the individuals served by your organization.

6. OTHER QUESTIONS

Why do we need to have a special meeting about overdose education and naloxone distribution (OEND) if I am on the HCS Coalition?
The goal of the meeting about OEND is to discuss the potential partnership between your organization and HCS, the requirements of the standing order agreement, and how OEND can be integrated into your organization's workflows. This goal is different from the typical agenda items of the HCS Coalition.

We were supposed to receive naloxone from KORE but haven't heard back about our request. What should we do with KORE? Tell them we are getting it from HCS?
The HCS team is partnering with KORE to expand overdose education and naloxone distribution. We can coordinate with your organization and KORE to ensure that you have the naloxone that your organization needs.

Will we have to obtain our naloxone from somewhere else when HCS is over?
Yes. We can work with you to plan around sustainability after HCS is over, but our funding to provide naloxone will end in December 2023.

NIH
HEAL
INITIATIVE

HEALing Communities Study
Kentucky

Naloxone Procedures for HCS Partner Agencies

Establishing a Standing Order Agreement

Naloxone is a prescription medication and must be dispensed under the order of a licensed practitioner. An HCS physician will sign a ***standing order agreement*** (SOA), which serves as an ongoing prescription to permit your agency to receive, store, and distribute naloxone. A representative of your agency will sign the SOA to indicate who at your agency will receive and distribute naloxone units and to establish the overdose education process you will follow for each unit distributed.

1. HCS will email a blank copy of the SOA
2. Read the SOA and determine who will receive and distribute naloxone for your agency (may be agency employees or HCS team members)

   HCS recommends providing a copy of these procedures to each person who will receive or distribute naloxone
3. Complete and sign the SOA either digitally or on paper
4. Email the SOA to **naloxoneHCS@uky.edu** or fax the SOA to **859-562-3356**
5. HCS will return a copy of your agency's SOA that is signed by our physician
6. Store a copy of the executed SOA at any site where the naloxone will be stored or distributed

   HCS recommends storing a copy of these procedures in the same place where the SOA is stored

Receiving Naloxone

HCS will dispense an initial allocation of naloxone to your agency as soon as possible following the execution of the SOA and will replenish the supply approximately quarterly. Quantities dispensed will be determined in partnership with members of the HCS team and will be based on anticipated need, current stock, and past distribution.

1. HCS will label each naloxone dose with your agency name and directions for use
2. HCS will deliver cases of naloxone either by a commercial parcel service (e.g., FedEx) or courier (an HCS team member)
3. An agency representative can acknowledge receipt by emailing **naloxoneHCS@uky.edu**
4. Store naloxone in a secure location at room temperature (between 59°F and 77°F)

Distributing Naloxone

Your agency may distribute HCS naloxone to any person who completes the required training. The agency is responsible for ensuring training was provided prior to the distribution of each unit of naloxone. The agency is also responsible for tracking and reporting how many units are distributed, the date on which each unit is distributed, and the demographics of individuals receiving naloxone.

1. Train each individual who is to receive naloxone

   Training requirement may be met by one of the following:
   1. Watching the HCS Educational Video
   2. Verbal review of the HCS Naloxone Brochure
   3. In-person training conducted by qualified agency representative (see SOA for required elements)
2. Document overdose education, collected demographic data, and naloxone distribution in the electronic survey or paper form.
3. Provide naloxone to trained individual
4. One to two times a week, an agency representative must submit paper form documentation of education and distribution to HCS via electronic survey.

Continue reading this manual for detailed electronic survey instructions

HCS Naloxone Program FAQs

What if our agency needs to change the list of representatives who can receive and distribute naloxone?

Contact the HCS Naloxone Program Manager by email at naloxoneHCS@uky.edu or fax to 859-562-3356. Provide the agency name, your name and title, and the changes you wish to make to the SOA. We'll update our copy and send back an addendum to store with your executed SOA.

What if our agency anticipates running out of naloxone before the scheduled quarterly replenishment?

You are able to request an off-schedule allocation of naloxone at any time. Contact your Implementation Facilitator to explain the situation and your anticipated needs, and we will work with you to maintain your supply of naloxone and adjust future replenishments.

Does HCS need the names of the people we distribute naloxone to?

HCS will never ask you for the names of the people receiving naloxone. We recommend protecting the private health information of the individuals receiving naloxone. We do ask you to collect some basic demographic information prior to naloxone distribution. An individual may decline to answer any of the demographic questions (excluding county of residence); simply leave those questions blank.

Key Contacts for HCS Naloxone Program

HCS Naloxone Coordinator

Email: naloxoneHCS@uky.edu
Phone: 859-562-3254
Fax: 859-562-3356

Trish Freeman

Email: trish.freeman@uky.edu
Phone: 859-323-1381

Doug Oyler

Email: doug.oyler@uky.edu
Phone: 859-562-3038

Naloxone Procedures
for HCS Partner Agencies

Reporting Distribution Using the Electronic Survey

NIH
HEAL
INITIATIVE

HEALing Communities Study
Kentucky

NIH HEAL INITIATIVE

HEALing Communities Study Kentucky

Recording Distribution of Naloxone in the Electronic Survey

Each unit of naloxone distributed by your agency must be recorded using your unique agency electronic distribution survey link. Demographics are also needed for each individual who is trained in overdose prevention and response. This information can be reported in real-time using your electronic distribution survey link or at a later point using the paper Training & Distribution form if internet access is not available at the time of distribution. Any paper forms should be submitted using the electronic distribution survey link as soon as internet access is available.

If you have questions about this process, please contact us at **naloxoneHCS@uky.edu**.

1. Access the electronic distribution survey

You can open your electronic distribution survey link using any device that has internet access.

Your agency's name should appear in the top left corner of the survey.

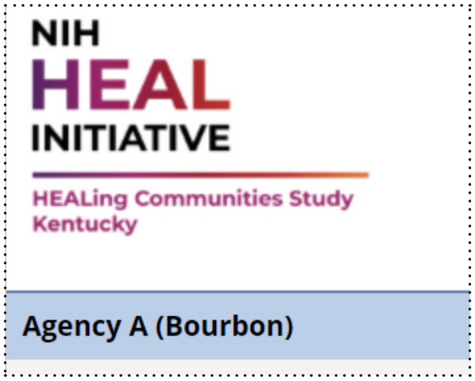

2. Fill out the survey

The first four questions (date, training, did the individual receive a unit of naloxone, and county of residence) are required.

While the other demographic questions are strongly encouraged to be answered by the individual, they are not required. Simply leave those questions blank if the individual chooses not to answer them.

3. Submit the survey

Once you have filled out the survey, select ***Submit*** at the bottom of the page. You will be redirected to a completion page where you can choose to record another distribution or leave the survey.

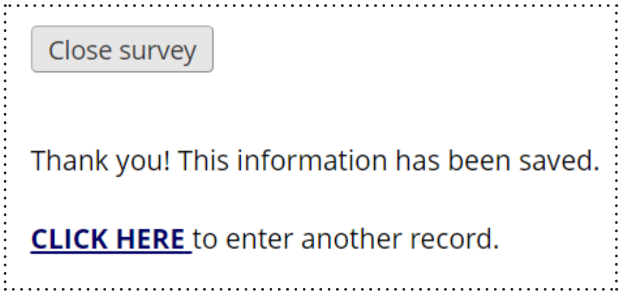

Repeat steps 2 through 3 for each unit of naloxone you distribute.

Don't forget to regularly enter and submit via the electronic survey any distributions recorded using the paper Training & Distribution form.

If you need your electronic distribution survey link, please contact your Implementation Facilitator or email naloxoneHCS@uky.edu.

NIH
HEAL
INITIATIVE

HEALing Communities Study
Kentucky

HCS Naloxone Training & Distribution Form

Each individual who is trained in overdose prevention and response must complete this form. A partner agency representative should complete the first three questions (date, type of training, and naloxone unit provided) and either ask individuals receiving naloxone the remaining questions or allow the individual to complete the form. Questions in the box are required.

Do not put the individual's name on this form. An individual may decline to answer any of the demographic questions (excluding county of residence); simply leave those questions blank. Information from the paper registration form must be entered into the electronic distribution survey to document the naloxone distribution.

Date: ____________________________

Please confirm you enter the correct date of distribution into the electronic survey

Type of Training: ☐ Video training ☐ Live session

Naloxone Unit Provided: ☐ Yes ☐ No

County of Residence: ____________________________

Ethnicity (Please choose one):

☐ Hispanic or Latino

☐ NOT Hispanic or Latino

Race (Please select all that apply):

☐ African American/Black

☐ White

☐ Asian

☐ American Indian/ Alaska Native

☐ Native Hawaiian or Other Pacific Islander

☐ Other, please specify:

____

Sex:

☐ Male

☐ Female

☐ Other

Age Range:

☐ Less than 18 years old

☐ 18-34 years old

☐ 35-54 years old

☐ 55 years of age or older

Have you ever overdosed?

☐ Yes

☐ No

☐ Not sure

Have you ever witnessed an overdose?

☐ Yes

☐ No

☐ Not sure

Overdose Education and Naloxone Distribution
FOLLOW-UP MEETING GUIDE

County: **Organization:**

Date of Meeting: **Start Time of Meeting:**

Before the Meeting, Prepare the Following:

A) Naloxone Report for Partners (should be sent to invitees before the meeting)

B) Names of Staff Listed on the SOA (gather list from SOA & insert list under #4 below)

C) Naloxone Inventory
Total Units Dispensed:
10% of Total Units Dispensed:
Total Units Distributed:
Net Inventory:
**Be sure to also enter inventory information on Table later in this Guide.*

D) Descriptions of OE and ND processes (see sections below)

1. Introductions
During the introductions in the Zoom meeting, document the names and titles of attendees (OEND liaison, any other attendees, etc.).

2. Overview of the Agenda
Here is an overview of today's agenda:
- Check in about the overdose education process
- Check in about the naloxone distribution process
- Discuss future naloxone shipments

3. Start the discussion about overdose education

[Before the meeting, the IF should insert a very brief summary of the overdose education process, including: (1) what type of training is being used for OE (e.g., HCS video or staff-led training), (2) who (which staff) are overseeing the OE part of the process, (3) whether the OE is being delivered in a group or individual setting, and (4) the general timing of when individuals receive OE. ***Provide this very brief summary to start the conversation.*** *For example: "So let's talk about how the overdose education process is going. When we met last, we discussed that overdose education would be offered to all individuals using the HCS video at the time of intake into your treatment program."*
NOTE: *If there is a lack of clarity around some of these aspects of the process, prepare before the meeting a short list of clarifying questions and then ask those clarifying questions during the meeting before moving on to the questions below.]*

How well is the overdose education process working? (*If not going well:* What revisions do you think are needed or what revisions have already been made?)

Have you encountered any barriers to providing overdose education? (*If yes:* What kinds of barriers have been encountered?)

Have there been any technology issues related to overdose education, if relevant? (*If yes:* What kinds of technology issues have come up?)

Are there any types of individuals who seem to be less likely to participate in the OE?

4. Transition to discussion about naloxone distribution

[Before the meeting, the IF should insert a very brief summary of the naloxone distribution process at the agency, including (1) how and when naloxone is being given to individuals, (2) who is responsible for distributing the naloxone (this may be the same person who is responsible for OE, but may not be), and (3) how the demographic questions are being collected (e.g., paper forms or directly in REDCap). ***Provide this very brief summary as a transition to talking about naloxone distribution. NOTE:*** *If there is a lack of clarity around some of these aspects of the process, ask clarifying questions before asking the questions below.]*

Did you encounter any barriers in receiving the first naloxone shipment or any barriers in documenting the receipt of it?

How well is the process for distributing naloxone working for individuals served by your agency? (*If not going well:* What revisions do you think are needed or what revisions have already been made?)

Here's the list of who is currently named on the Standing Order Agreement for OEND: [insert list below]

Have there been any changes to individuals conducting OEND?
[If so, the IF should provide names that should be added or removed from the SOA to the Naloxone Coordinator (naloxoneHCS@uky.edu)]

What, if any, challenges have there been in documenting the distribution and demographic questions in the electronic survey?

Has there been any feedback from people as they have received naloxone? What has that feedback been?

5. Naloxone supply

[Before the meeting, the IF should insert below how many doses were included in the first shipment, and estimated quarterly shipment size (3 times the difference of the first shipment and the estimated existing inventory, rounded to the nearest 12 units).]

Did you receive the report that we sent about naloxone doses that have been distributed so far?

Our data is showing that your agency has distributed ______ units. Is that correct?

[IF should share her/his screen to show the report if the OEND Liaison is attending via Zoom. If there is a discrepancy in the number distributed, the IF should ensure the agency has recorded all distribution data in the electronic survey. The agency should be encouraged to search their site for units that have not been accounted for]

You should have ___ units on-hand. Is that number correct?
[If not, the IF should ensure the agency has recorded all distribution data in the electronic survey The IF should also attempt to reconcile the discrepancy with the agency, potentially asking about units that may have been distributed but not documented and units that could have been lost, damaged, or administered.

To determine if the difference meets the HCS definition of a Discrepancy, use the table below]

HCS Data on Net Inventory	
Subtract Agency's Self-Reported Inventory	-
Difference in Inventory	
10% of Total Dispensed (see page 1)	

If the number in the row for 10% of Total Dispensed is larger than the number in Difference in Inventory and the Difference in Inventory is greater than 12 units, there is a Discrepancy that must be resolved. The IF should determine if the agency has not been entering data or has distributed naloxone without documenting it. NOTE: Replenishment will not be sent until discrepancy is resolved; see SOP for details.

The first shipment included ____ units. Does the shipment amount seem appropriate for a month? We'd like to ship naloxone on a quarterly basis, which would be ____ units to cover distribution over 3 months. Does that seem like too much, too little, or just right?

6. Wrap-up

What could we do to make overdose education and naloxone distribution easier or more efficient for your agency?

[The IF should then summarize any "next steps" that were identified during the discussion.]

Thank you for your time today, and also for all your work to deliver naloxone to individuals served by your agency. We greatly appreciate your time and effort. I'll send an email before our next check-in. If you need additional naloxone units, please email me and I will work with our team toward fulfilling the request.

End Time of Meeting: _________________

NIH HEAL INITIATIVE
HEALing Communities Study Kentucky

Naloxone Report: Units Shipped and Distributed
Organization: Name
Date of First Naloxone Shipment: Month Day, Year
Date of Report: Month Day, Year

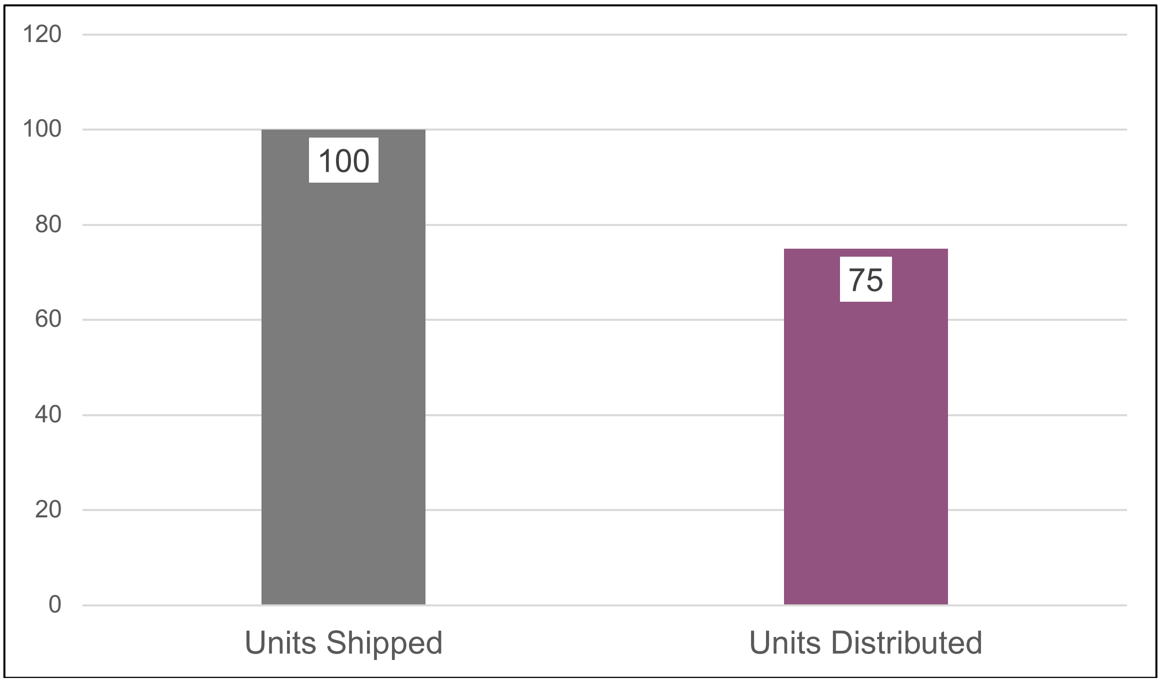

Questions about overdose education and naloxone distribution, including changes to future shipments can be sent to [IF name] (IF email address).

Overdose Education and Naloxone Distribution

SUSTAINABILITY MEETING GUIDE

County:________________ **Organization:____________________**

Date of Meeting:__________________ **Start Time of Meeting:_____________**

Tasks Before the Meeting

Before the meeting, the IF should draft a very brief summary of the OEND process, including:

- *The basics of how OEND is implemented at the agency (e.g., staff involved, the clients/patients who are the focus of the OEND process, the primary ways that OE is delivered)*

Then the IF should tailor the OEND Summary Report using information in the most recent naloxone distribution counts for the agency. For the OEND Summary Report, please complete the following:

- *Insert the brief summary of the OEND process*
- *Insert numbers from the Excel file into the yellow highlights (remove the highlights once complete)*

Review the distribution numbers—if you have any concerns about the data, please coordinate with the Naloxone Coordinator before sharing the report with the agency

- *Be sure to insert the current inventory number (see two yellow highlights in the meeting guide)*

Tasks For the Meeting

1. Document Attendees

During the start of the Zoom meeting, document the names and titles of attendees (OEND liaison, any other attendees, etc.). Document the meeting start time.

2. Set the Agenda

Here is an overview of today's agenda:

- Discuss and reflect on the OEND partnership between your agency and HCS
- Discuss how OEND could be sustained at the agency

3. Start the Discussion about the OEND Partnership

> *To start the discussion, the IF will share that very brief summary that was drafted before the meeting and the Summary Report. After sharing the summary, the IF will lead a discussion about the partnership.*

As you know, your agency implemented a model of OEND where….[provide summary of OEND here].

In terms of your agency's distribution, your agency ….[provide summary of their distribution based on the OEND Summary report here].

Be sure to screen share the agency's OEND Summary Report

Be sure to thank them for the naloxone that they distributed during the study

Looking back at the experience of implementing OEND at your agency, what do you think has worked well?

What, if any, significant barriers or challenges have you had in implementing OEND at your agency?

- *If there were significant barriers:* To what extent do you think you were able to overcome those barriers? What helped you overcome those barriers?

How could HCS improve its support for OEND?

What advice would you have for other agencies (that are similar to yours) about implementing OEND?

4. Start the Discussion about Sustainability

How important do you think it is for your agency to continue implementing OEND with the clients/patients that you serve?

- Can you share some of the main reasons for why it's [important/somewhat important/not very important) for your agency?

How interested is your agency in continuing to implement OEND after the partnership with HCS ends?

- ***If agency is interested in sustaining, skip to next page.***
- ***If agency is not interested in continuing to implement OEND after HCS, share the following:***
    - HCS can leave the remaining naloxone with your agency to continue OEND until it runs out. Our record show you should have XX units on hand. Is that accurate?
    - Would you like to continue OEND until the supply of naloxone has run out?
        - *If they choose to continue OEND until the supply is gone, share with the agency that:*
            - They will need to continue to implement OEND in a way that adheres to the standing order agreement.
            - HCS will still need the agency to upload distribution data & demographic data for these units

        - The HCS Naloxone Coordinator will ask for any remaining naloxone to be returned to us if it is nearing its expiration date
    - *If they want to return the naloxone to us:*
        - Ask for a point of contact to coordinate a day/time for HCS staff to come pick up the units
- Ask if they would like to keep the computer tablet--We can leave the computer tablet(s) in case any staff want to continue to use the overdose education videos
    - *If they want to return the tablet to us:*
        - Ask for a point of contact to coordinate a day/time for HCS staff to come pick up the units
- ***If agency has a prescriber (MOUD agency, health care), please share the following:***
    - The Kentucky Opioid Response Effort, or KORE, is rolling out a prescription co-pay card for patients with commercial insurance who have co-pays when they fill a prescription for naloxone. The focus is commercial insurance, not patients with Medicaid or other federal insurance coverage. But patients with commercial insurance can give the card to the pharmacist, which will reduce their co-pay by up to $60. Would you have interest in us sending you a short pamphlet that you can share with your prescribers about this co-pay program?
        - If yes: How many prescriber pamphlets would you like us to send to you? ______
        - And would you like us to send some of these co-pay cards for patients, and if so, how many?_____cards
- *Then, close the meeting by:*
    - Expressing gratitude for the partnership
    - Asking if they have any additional feedback or questions

<u>For all agencies interested in continuing OEND</u>:

HCS wants to support agencies as best we can but we also need to start planning for longer term sustainability. Some of the resources that we can provide:

- A final naloxone shipment that should cover approximately 90 days of distribution based on your recent distribution data
    - HCS will still need your agency to upload distribution data & demographic data for these units
    - The HCS Naloxone Coordinator will ask for any remaining naloxone to be returned to us if it is nearing its expiration date
- We will leave the computer tablet(s) with the overdose education videos
- We will email links to any future overdose education videos that we post to YouTube

- We will share the PDF file of the naloxone brochure so that the agency can print additional copies as needed, and we'll send updates if we make changes to the brochure

Would these resources be helpful?

Our records show that you have XX units on hand. Is that accurate?

Based on your previous distribution, we have estimated that the final shipment would be ______units. Does that sound like it would cover your agency for about 3 months?

> *Note: If you'd rather discuss the final shipment after talking about KORE's naloxone program (below), feel free to do so.*

Finally, let's talk about an option for sustaining your supply of naloxone.

The Kentucky Opioid Response Effort (KORE) is a key partner with HCS and has the ability to support many of the agencies that have implemented OEND through HCS. KORE has received federal funding to support OEND, and KORE has partnered with the Kentucky Pharmacists Association to order and ship naloxone to partner agencies at no-cost to the partner agency. Partner agencies then send data on the units of naloxone that they have distributed back to KPhA. Basically, the partnership is quite similar to how OEND has worked during HCS.

> *Note: You can share with the agency that we're trying to stagger the transition of agencies to KORE naloxone—if we did all of the agencies at once, there might be delays, so that is why we are asking them to start the transition soon (so there is no gap between the final shipment and their first KORE shipment).*

There's an online form for requesting naloxone through KORE's partnership with the KPhA. Would you like for me to send you a link after the meeting?

> **If yes:** Great! I'll do that right after this meeting, but in the meantime, I'll place the link in the chat. (www.kphanet.org/KORE).
>
> A few things to know:
>
> The form will ask about staff training. Because of our partnership where you and your staff have been trained in OEND, there are no additional training requirements for KORE's naloxone program. The process for distributing naloxone should remain the same, meaning that your organization can plan to continue distributing naloxone in the same way you are doing now and with the same protocol for providing overdose education alongside the naloxone distribution. However, if you do feel like your agency needs additional training, you can request training on the form in the section labeled, "Training Request." You can just leave that section blank if you do not have any training needs.
>
> Also, the form will ask how much naloxone you have already and how much you are requesting, so you'll want to be ready with those numbers. We recommend

you request the equivalent of a 2-month supply of naloxone each time you order. You might not be reminded to restock when your supply is low, so please plan to monitor your supply and order before you run out.

Finally, I'll also send you the contact information for Dr. Jody Jaggers. He is the Director of Pharmacy Public Health Programs for the Kentucky Pharmacists Association, and Dr. Jaggers oversees KORE's naloxone partnerships with agencies. (email: KORE@kphanet.org)

Do you need anything else from us to support your ongoing OEND program?

5. Close the meeting

- Express gratitude for the partnership
- Summarize any action items/next steps discussed
- Ask if they have any additional feedback or questions

End Time of Meeting:

Post Meeting Checklist

Does the agency want to sustain OEND?

___Yes
___No

Have you sent the OEND Summary Report to the meeting attendees?
___Yes
___No

Have you sent via REDCap the request for the agency's final quarterly shipment?
___Yes
___No (agency doesn't need/want more naloxone)

If OEND will be sustained, have you sent the link to KORE/KPhA's website (www.kphanet.org/KORE) and Dr. Jody Jagger's KORE email address (KORE@kphanet.org) to the agency?
___Yes
___No
___Not applicable (OEND will not be sustained)

If OEND will not be sustained, does agency have HCS resources (e.g., naloxone, tablets) to return to us at this time?
___Yes

___No
___Not applicable (OEND will be sustained)
If yes, Please make arrangements for an HCS staff member (Community Coordinator or Prevention Specialist) to pick up these resources and to notify the HCS Naloxone Coordinator once it has been completed.

NIH HEAL INITIATIVE
HEALing Communities Study Kentucky

University of Kentucky

Overdose Education and Naloxone Distribution (OEND) Summary Report

[Agency Name – County Name]
[Date Prepared]

Current Model of OEND

[Insert summary here]

Naloxone Distribution to Date

Your first recorded distribution was **[date of first distribution]**. Since that time, you have distributed [**total number]** units, for a monthly average of [##] units per month. A 6-month trend of your naloxone distribution is below:

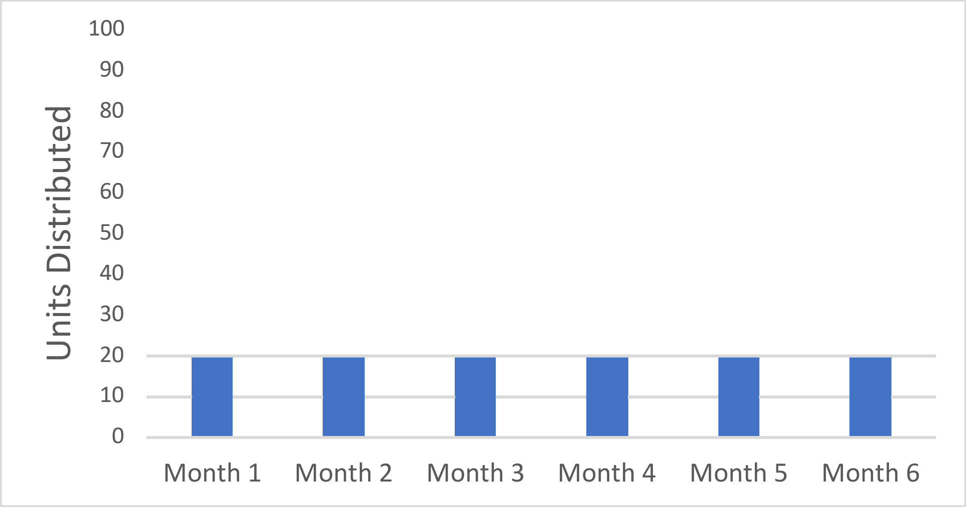

Options to Sustain OEND

The estimated annual cost of supporting your OEND efforts, using a public health price of $47.50 per unit, is **[$price x units per year]**. Other efforts that could potentially help support OEND include:

- Partnership with the **Kentucky Opioid Response Effort (KORE)** by visiting https://www.kphanet.org/kore.
- Your county Agency for Substance Abuse Policy (ASAP) Board.
- Discussion with internal health care providers with prescriptive authority (e.g., physicians, nurse practitioners, and physician assistants). Naloxone is available at most pharmacies and is covered by most insurance providers with little-to-no copay.

Educational Materials

Overdose education materials from the study are available on YouTube in English, Spanish, Arabic, and Swahili and can be accessed using the QR code. We can provide a printable PDF copy of the naloxone educational brochure to supplement on-site education as well.
